# A J Domain Protein Functions as a Histone Chaperone to Maintain Genome Integrity and the Response to DNA Damage in a Human Fungal Pathogen

**DOI:** 10.1128/mbio.03273-21

**Published:** 2021-12-21

**Authors:** Linda C. Horianopoulos, Christopher W. J. Lee, Kerstin Schmitt, Oliver Valerius, Guanggan Hu, Mélissa Caza, Gerhard H. Braus, James W. Kronstad

**Affiliations:** a Michael Smith Laboratories, Department of Microbiology and Immunology, University of British Columbia, Vancouver, British Columbia, Canada; b Institut für Mikrobiologie & Genetik, Georg-August-Universität, Göttingen, Germany; Institut Pasteur

**Keywords:** histone chaperone, J domain proteins, DNA damage, hydroxyurea, transcriptional response, histone chaperone

## Abstract

Histone chaperoning ensures genomic integrity during routine processes such as DNA replication and transcription as well as DNA repair upon damage. Here, we identify a nuclear J domain protein, Dnj4, in the fungal pathogen Cryptococcus neoformans and demonstrate that it interacts with histones 3 and 4, suggesting a role as a histone chaperone. In support of this idea, a *dnj4Δ* deletion mutant had elevated levels of DNA damage and was hypersensitive to DNA-damaging agents. The transcriptional response to DNA damage was also impaired in the *dnj4Δ* mutant. Genes related to DNA damage and iron homeostasis were upregulated in the wild-type strain in response to hydroxyurea treatment; however, their upregulation was either absent from or reduced in the *dnj4Δ* mutant. Accordingly, excess iron rescued the mutant’s growth in response to DNA-damaging agents. Iron homeostasis is crucial for virulence in C. neoformans; however, Dnj4 was found to be dispensable for disease in a mouse model of cryptococcosis. Finally, we confirmed a conserved role for Dnj4 as a histone chaperone by expressing it in Saccharomyces cerevisiae and showing that it disrupted endogenous histone chaperoning. Altogether, this study highlights the importance of a JDP cochaperone in maintaining genome integrity in C. neoformans.

## INTRODUCTION

Cryptococcus neoformans is an opportunistic fungal pathogen that can survive and proliferate in the environment as well as within mammalian hosts (1). Maintenance of genomic integrity in both of these niches is required so that genetic information can be faithfully transmitted to progeny (2). Chromatin regulatory factors and histone chaperones ensure genomic stability during DNA replication and transcription. Additionally, specialized DNA repair proteins (e.g., Rad proteins) respond to DNA lesions caused by DNA damage from stresses such as reactive oxygen species encountered in the host (3, 4). Together these groups of proteins maintain genomic integrity during routine growth and under stressful conditions.

The histone chaperones and modifying proteins influence genomic stability at the level of chromatin (3), and several have importance in fungal pathogens. For example, the histone acetyltransferase Rtt109 acetylates histone 3 to regulate transcriptional responses to stress. This protein is required for the proper regulation of morphology, progression through life cycle stages, and the ability to cause disease in several fungal pathogens, including Beauveria bassiana, Magnaporthe oryzae, and Candida albicans (5–7). Other histone acetylases, such as Hat1 in C. albicans and Gcn5 in C. neoformans, also play roles in fungal virulence (8, 9). Similarly, histone deacetylases contribute to fungal virulence, and this finding is of considerable interest because inhibition of these enzymes acts synergistically with azole antifungal drugs *in vitro* (10–13). Furthermore, several protein chaperones involved in chromatin dynamics (Hir1, Msl1, and Cac2) and a core histone chaperone (Asf1) are required for morphological changes and development that contribute to pathogenesis-related phenotypes (e.g., capsule elaboration in C. neoformans and hyphal formation in C. albicans) (14–17). Overall, these studies indicate the importance of histone chaperones and modifying enzymes in diverse fungal pathogens.

Several DNA repair proteins also play important roles in fungal pathogens, often acting together. For example, the Rad53 and Chk1 kinases were shown to cooperatively regulate virulence in C. neoformans (2). Interestingly, a number of genes encoding functions for radiation resistance (18), hypermutator phenotypes (19, 20), and mitochondrial DNA repair (21) contribute to aspects of genomic integrity but are dispensable for virulence. These findings highlight the complexity of the roles of DNA repair pathways in fungal proliferation in host tissue. To address this complexity, it is important to uncover novel elements that support genome integrity and to understand its maintenance in fungi. Toward this goal, we characterized the role of a dual heat shock and histone chaperone, Dnj4, in the response to DNA damage in C. neoformans.

## RESULTS

### Dnj4 is an ortholog of DNAJC9, a nuclear J domain protein in humans.

**FIG 1:**
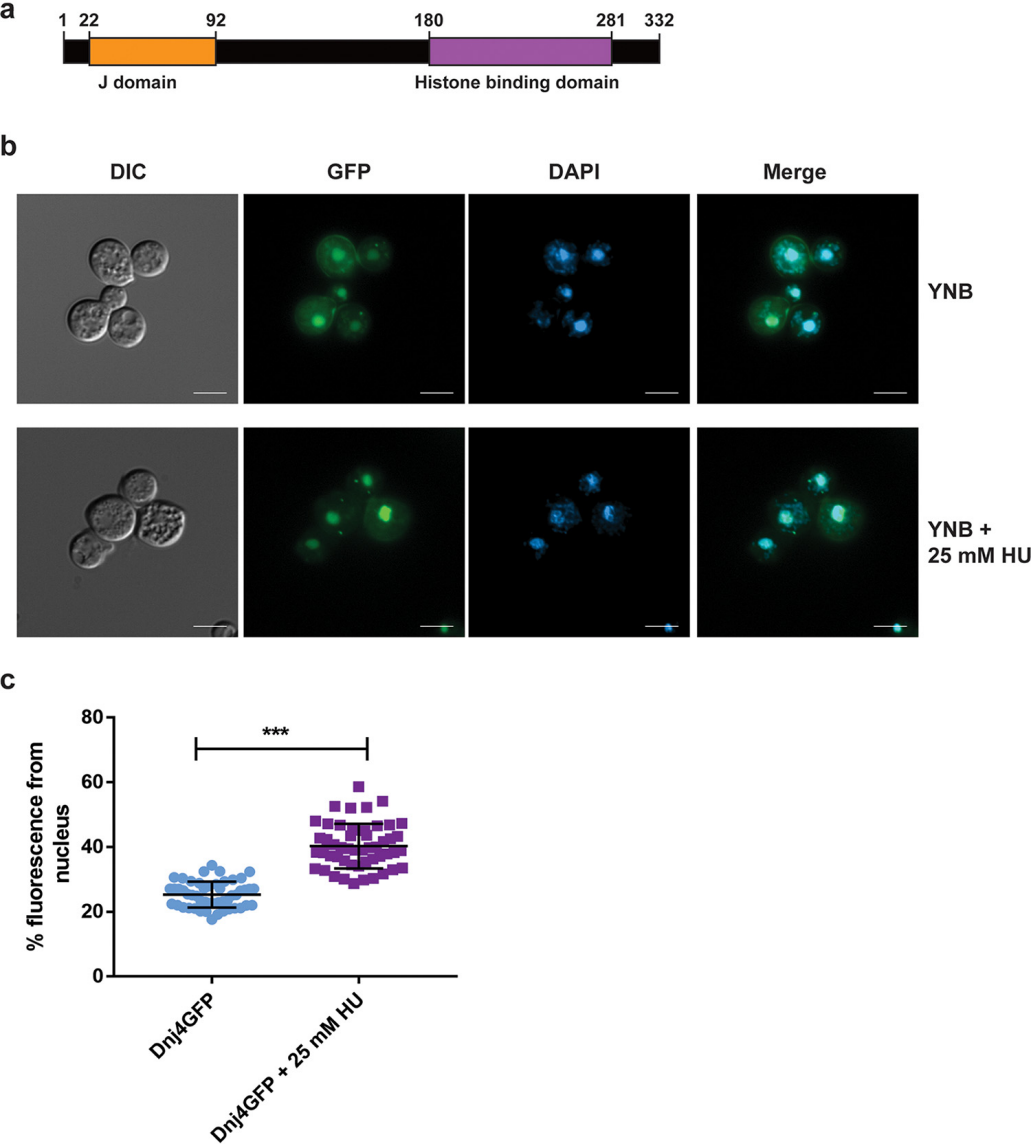
Dnj4 is a nuclear localized J domain containing protein. (a) A schematic of Dnj4 indicating the J domain and the histone binding domain (HBD) inferred from homology to the HBD determined in DNAJC9. (b) Dnj4-GFP was found to primarily localize to the nucleus as observed through costaining with DAPI. However, upon hydroxyurea treatment, the proportion of GFP signal in the nucleus was increased, suggesting increased nuclear recruitment in response to DNA damage (bar, 5 μm). (c) Quantification of the proportion of total fluorescence from the nucleus for 50 cells is shown as individual dots with the mean and standard deviation indicated with horizontal lines. Statistical significance was determined using an unpaired *t* test (***, *P* < 0.005).

The Dnj4 protein has a conserved N-terminal J domain and is therefore predicted to be a cochaperone (Fig. 1a). In support of this prediction, we found that Dnj4 was an ortholog of the human cochaperone DNAJC9 (22) and to proteins in a number of fungal species, with the exception of Saccharomyces cerevisiae. We examined the domain structure of Dnj4 and generated a maximum likelihood tree with DNAJC9 and orthologous amino acid sequences obtained from UniProt for several fungi. This analysis revealed that the closest ortholog was from another basidiomycete pathogen, Ustilago maydis (see Fig. S1a in the supplemental material). With regard to domain structure, the recent characterization of DNAJC9 identified a C-terminal histone binding domain (HBD); this domain was largely conserved across species, although an insertion was observed in the alignment of some of the fungal sequences (Fig. S1b) (22).

10.1128/mbio.03273-21.1 FIG S1 C. neoformans Dnj4 has conserved domains with human DNAJC9. (a) The maximum likelihood tree comparing Dnj4, DNAJC9 from both *M. musculus* and H. sapiens, and other fungal orthologs determined from FungiDB is shown with a schematic of each polypeptide indicating the location of the J domain and histone binding domain (HBD) based on similarity to the recently characterized HBD of human DNAJC9. This tree was constructed by ClustalW alignment of full-length amino acid sequences retrieved from UniProt and the Le Gascuel amino acid replacement matrix was used to build the tree. The bootstrap values from 500 bootstrap trees are indicated at the nodes in the tree. (b) An alignment of the putative HBDs for the DNAJC9 orthologs. Asterisks indicate the residues which interact with histones in DNAJC9. Download FIG S1, SVG file, 0.3 MB. Copyright © 2021 Horianopoulos et al. 2021 Horianopoulos et al. https://creativecommons.org/licenses/by/4.0/ This content is distributed under the terms of the Creative Commons Attribution 4.0 International license.

The roles of cochaperones in the J domain family are known to be influenced by their subcellular localizations, as they increase the local concentration of Hsp70s and direct the activity of these chaperones toward processes in their specific locations (23). In this context, we created a strain expressing a Dnj4-green fluorescent protein (GFP) fusion protein and observed its localization to obtain clues about its potential functions. Costaining with 4′,6-diamidino-2-phenylindole (DAPI) revealed that Dnj4-GFP was located primarily in the nucleus but with some signal from the cytoplasm (Fig. 1b). The human ortholog, DNAJC9, has been reported to change localization from the nucleus to the plasma membrane upon heat shock in A549 epithelial cells (24). However, in C. neoformans Dnj4-GFP remained primarily in the nucleus upon heat shock at both 37°C and 42°C (Fig. S2). Interestingly, the addition of the DNA damaging agent hydroxyurea (HU) increased the proportion of GFP signal from the nucleus, suggesting that the nuclear concentration of Dnj4 increased upon genotoxic stress through either altered localization, increased expression, or a combination of these factors (Fig. 1c).

10.1128/mbio.03273-21.2 FIG S2 Dnj4-GFP is primarily nuclear localized regardless of elevated temperature. Colocalization of GFP-tagged Dnj4 with the nuclear stain DAPI is shown. The nuclear localization is maintained at elevated temperature (bar = 5 μm). Microscopy images are representative of 30 images captured. Download FIG S2, SVG file, 0.08 MB. Copyright © 2021 Horianopoulos et al. 2021 Horianopoulos et al. https://creativecommons.org/licenses/by/4.0/ This content is distributed under the terms of the Creative Commons Attribution 4.0 International license.

### Dnj4 interacts with histones 3 and 4.

**FIG 2:**
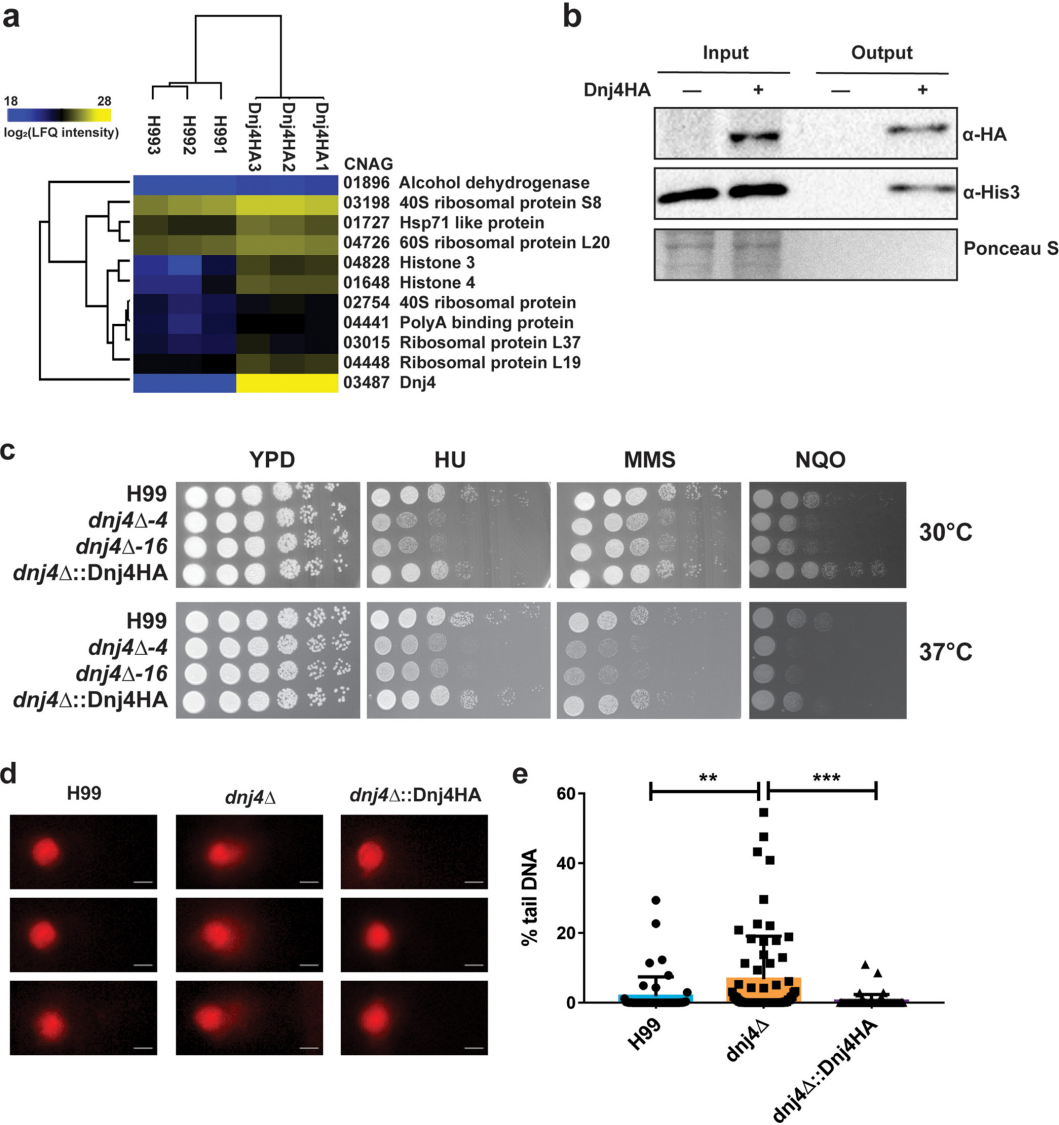
Dnj4 interacts with histones and impacts genome integrity. Proteins that coimmunoprecipitated with Dnj4HA as bait using anti-HA magnetic beads were identified using mass spectrometry. (a) The significantly enriched proteins were identified using a one-sided *t* test with a false discovery rate of 0.05 in Perseus. These proteins are shown in a heat map based on LFQ intensity. (b) The interaction with histones was confirmed by immunoblotting. An interaction with histone 3 was observed in three repeats of the experiment. (c) Spot assays of serially diluted wild-type (H99), two independent *dnj4Δ* mutants, and complemented (*dnj4Δ*::Dnj4HA) strains on YPD plates supplemented with the DNA-damaging agents hydroxyurea (HU; 25 mM), methyl methanesulfonate (MMS, 0.03%), or 4-nitroquinoline-1-oxide (NQO, 0.1 μg/ml) are shown. Plates were incubated at 30°C or 37°C for 2 days. (d) Representative images of the comets scored showing increased tail DNA for the comets generated from the mutants lacking *DNJ4* (bar, 2 μm). (e) The percentage of DNA in the tail portion of comets produced from spheroplasts were measured for 50 comets generated for each strain and are indicated as individual dots with the bar representing the means and error bars representing standard deviations. Statistical significance was determined using a one-way ANOVA with Tukey’s multiple comparisons (**, *P* < 0.01; ***, *P* < 0.005).

To further characterize the role of Dnj4, we identified candidate interacting partners of Dnj4HA using affinity purification and mass spectrometry (AP-MS). A total of 314 proteins were identified by MS after filtering out contaminants, reverse peptides, and proteins that did not appear in all three replicates of the Dnj4HA eluate. We found 11 significantly enriched proteins, including histones 3 and 4 as the most enriched proteins besides Dnj4 (Fig. 2a, Table S1). Furthermore, Hsp71 (CNAG_01727) was significantly enriched in the Dnj4HA eluate. The interaction with histones was of particular interest, as Dnj4 was observed to localize in the nucleus. Therefore, we confirmed the interaction with histones by immunoblotting against Dnj4HA and histone 3 after coimmunoprecipitation with anti-hemagglutinin (HA) magnetic beads (Fig. 2b). This is consistent with the recent findings in humans that DNAJC9 interacts with histones and the human Hsp71 ortholog, Hsc70 (22). Altogether, these results suggest a role for Dnj4 in histone chaperoning.

10.1128/mbio.03273-21.7 TABLE S1 Proteins detected through AP-MS. The Δlog2(LFQ intensity) is the average change in label-free quantification (LFQ) intensity from 3 replicates between the pulldown with the Dnj4-HA tagged strain and the WT control. The *P* value is derived from a one-sided *t* test (FDR, 0.05) performed in Perseus and the significance based on this test is indicated in the first column. Download Table S1, PDF file, 0.08 MB. Copyright © 2021 Horianopoulos et al. 2021 Horianopoulos et al. https://creativecommons.org/licenses/by/4.0/ This content is distributed under the terms of the Creative Commons Attribution 4.0 International license.

### Mutants lacking *DNJ4* are hypersusceptible to DNA damaging agents.

We next examined the role of Dnj4 in the response to DNA damage given its nuclear localization and interaction with histones. Two independent deletion mutants lacking *DNJ4* were generated using biolistic transformation and confirmed by genomic hybridization (Fig. S3). The *dnj4*Δ-16 mutant was complemented with a Dnj4-HA fusion protein at the native locus (*dnj4*Δ::Dnj4HA) using biolistic transformation. The *dnj4*Δ mutants were found to be hypersusceptible to the ribonucleotide reductase inhibitor HU, which results in nucleotide depletion, as well as the alkylating agent methyl methanesulfonate (MMS) and the DNA damaging agent 4-nitroquinoline-1-oxide (NQO), which both act as mutagens. These growth defects were observed at both 30°C and 37°C and were rescued in the complemented strain (Fig. 2c). This hypersensitivity suggests that Dnj4 contributes to genomic stability or to DNA damage repair. These mutants were not broadly found to have other growth defects and showed no hypersensitivity to oxidative stress, cell wall stress, or antifungal drugs (Fig. S4).

10.1128/mbio.03273-21.3 FIG S3 Southern hybridization confirmation of the genotype of the *dnj4Δ* strains. DNA from the indicated strains was extracted and digested with KpnI and EcoRV at the indicated dashed lines, and genomic hybridization was performed using a DIG-labeled DNA probe (SP). The probe detected fragments of 1,908 bp in the wild type and 4,532 bp in both deletion mutants. A DIG-labeled DNA ladder is also shown. Download FIG S3, SVG file, 0.07 MB. Copyright © 2021 Horianopoulos et al. 2021 Horianopoulos et al. https://creativecommons.org/licenses/by/4.0/ This content is distributed under the terms of the Creative Commons Attribution 4.0 International license.

10.1128/mbio.03273-21.4 FIG S4 Mutants lacking *DNJ4* are not broadly susceptible to other inhibitors. The mutants lacking *DNJ4* were assayed for general growth defects compared to the wild-type and complemented strains and showed no hypersensitivity to oxidative stress (H_2_O_2_, tert-butyl hydroperoxide [tBOOH], and menadione), cell wall stress (caffeine and Congo Red [CR]), or antifungal drugs (fluconazole and miconazole). Spot assays shown are representative of three replicates. Download FIG S4, SVG file, 0.8 MB. Copyright © 2021 Horianopoulos et al. 2021 Horianopoulos et al. https://creativecommons.org/licenses/by/4.0/ This content is distributed under the terms of the Creative Commons Attribution 4.0 International license.

### In the absence of *DNJ4*, C. neoformans displays increased levels of DNA damage.

Since Dnj4 was found to interact with histones, and the human ortholog DNAJC9 participates in histone supply and deposition during replication and transcription, (22) we hypothesized that Dnj4 may be required to maintain genomic integrity. To test this hypothesis we employed an alkaline comet assay (25) with C. neoformans spheroplasts and measured the percentage of DNA in the comet tail to assess the degree of DNA damage in cells. The comets generated in the *dnj4Δ* mutant had significantly more DNA in the comet tail relative to those generated from the wild-type (WT) or complemented strains (Fig. 2d and e). This finding indicates that mutants lacking *DNJ4* have higher levels of DNA damage than the WT and complement. This is also supported by the hypersensitivity of these mutants to DNA damage-inducing agents.

### Dnj4 is required for the transcriptional response to DNA damage.

**FIG 3:**
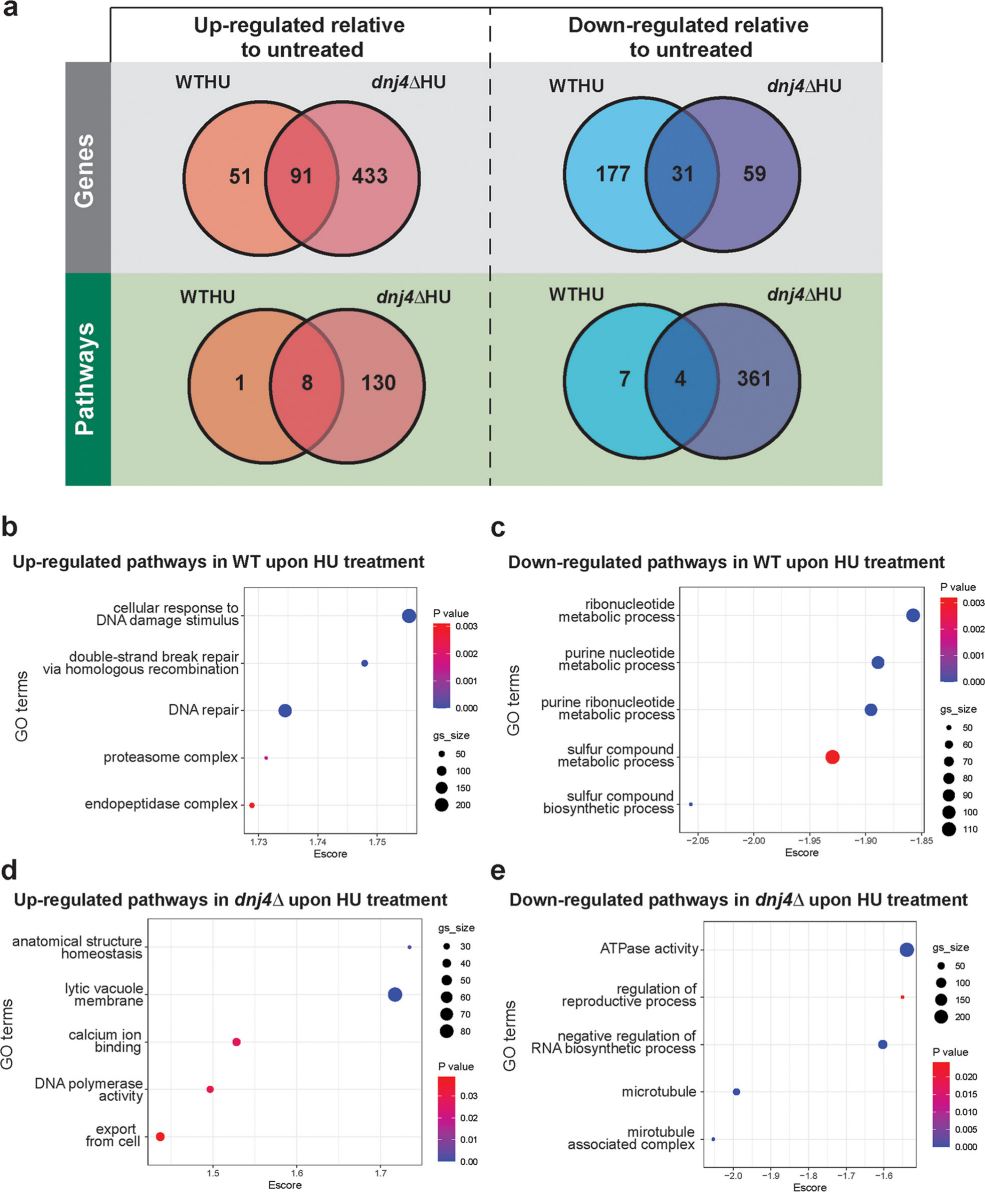
Loss of *DNJ4* impacts the transcriptional response to hydroxyurea. RNA was collected from three biological replicates of wild-type (WT) and mutant (*dnj4Δ*) strains after 1 h of hydroxyurea (HU) treatment and from untreated controls. RNA sequencing was used to assess changes in the transcriptional response to HU treatment between strains. (a) Venn diagrams comparing the significantly differentially regulated genes and pathways upon HU treatment for the WT and *dnj4Δ* deletion mutant. For the gene Venn diagrams, significance was determined using a *P* value cutoff of 0.05 and a minimum 2-fold change for upregulated genes and 2-fold change for downregulated genes. For the pathway Venn diagrams, significant enrichment was determined using a *P* value cutoff of 0.05 and false discovery rate of 0.25. (b to e) The significantly enriched pathways were determined using an enrichment score in GSEA and the top 5 hits of upregulated (b and d) and downregulated (c and e) pathways for each strain upon HU treatment are shown (gs_size, gene set size; GO, gene ontology; Escore, enrichment score).

Since Dnj4 plays a role in the response to DNA damage, we characterized the transcriptional response of the WT and the *dnj4Δ* deletion strains to HU, which depletes cellular levels of nucleotides and results in induction of the DNA damage stress responses (26, 27). Transcriptome sequencing (RNA-Seq) was conducted on HU-treated and untreated cells of both strains. In response to HU treatment, 142 genes were significantly upregulated and 208 genes were significantly downregulated in the WT. In the *dnj4Δ* mutant, 524 genes were significantly upregulated and 90 genes were significantly downregulated (Fig. 3a, Data Set S1). Pathway analysis was then conducted using all genes to better understand the coordination of the transcriptional response. In response to HU treatment, the WT had only 9 pathways significantly upregulated and 11 pathways significantly downregulated. In contrast, the *dnj4Δ* mutant had 138 upregulated pathways and 365 downregulated pathways. In the WT, the top upregulated pathways were related to DNA repair (cellular response to DNA damage stimulus, double-strand break repair via homologous recombination, and DNA repair) and also to protein recycling (proteasome and endopeptidase complexes). Surprisingly, the top upregulated pathways in the *dnj4Δ* mutant in response to HU treatment were largely unrelated to DNA dynamics with the exception of DNA polymerase activity. In response to HU treatment, metabolic pathways were the top downregulated pathways in the WT, whereas pathways related to microtubules, ATPase activity, regulation of reproductive processes, and negative regulation of RNA biosynthetic processes were downregulated in the *dnj4Δ* mutant (Fig. 3b). The enrichment of fewer pathways in the WT was surprising at first; however, this may represent a more coordinated response, particularly because the pathways upregulated are those involved in the DNA repair pathways, which would be required to respond to DNA damage induced by HU treatment. In contrast, the mutant had many pathways up- and downregulated, and this finding indicates a dysregulated transcriptional response.

10.1128/mbio.03273-21.6 Data Set S1 RNA-Seq data for the wild-type strain H99 compared with the *dnj4Δ* mutant with and without treatment with hydroxyurea (HU). Download Data Set S1, XLSX file, 1.2 MB. Copyright © 2021 Horianopoulos et al. 2021 Horianopoulos et al. https://creativecommons.org/licenses/by/4.0/ This content is distributed under the terms of the Creative Commons Attribution 4.0 International license.

**FIG 4:**
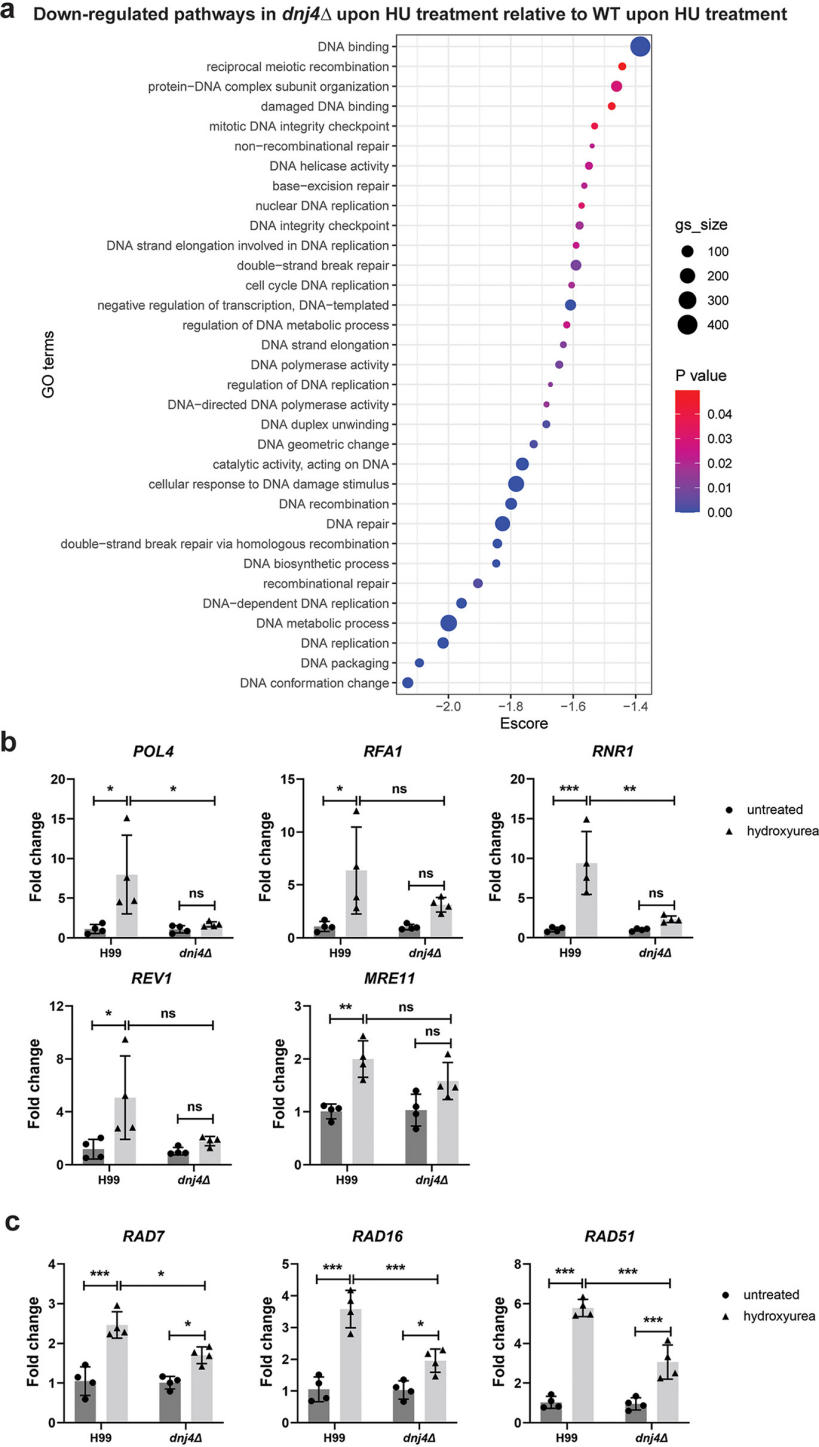
Impact of *DNJ4* on the regulation of the DNA damage response to hydroxyurea (HU) treatment. (a) In the mutant lacking *DNJ4* (*dnj4Δ*) treated with HU, pathways related to DNA damage and repair were downregulated relative to the wild type (WT) treated with HU (gs_size, gene set size; GO, gene ontology; Escore, enrichment score). (b and c) RT-qPCR confirmation of the upregulation of the indicated genes related to the DNA damage response (b) and radiation repair (c). The means from four biological replicates are shown, and the error bars show the standard deviations. Statistical significance was determined using a two-way ANOVA and Tukey’s multiple comparisons (ns, not significant; *, *P* < 0.05; **, *P* < 0.01; ***, *P* < 0.005).

DNA damage response and repair genes are upregulated in response to HU in S. cerevisiae (28, 29). Therefore, it was surprising that the top upregulated pathways in the *dnj4Δ* mutant were generally unrelated to DNA damage and repair pathways. We performed a comparison of the RNA-Seq data between strains to assess the enrichment of pathways related to DNA damage in the WT and mutant samples in response to HU treatment. Many pathways related to DNA damage were significantly downregulated in the *dnj4Δ* mutant compared to the WT, suggesting that the DNA damage response was decreased upon loss of Dnj4 (Fig. 4a). This impaired response was confirmed for several genes using quantitative reverse transcription-PCR (RT-qPCR). Among the DNA repair genes upregulated in response to HU, several were not significantly upregulated in the *dnj4Δ* mutant, including genes encoding DNA polymerase IV (*POL4*), replication factor A1 (*RFA1*), deoxycytidyl transferase (*REV1*; involved in repair of abasic sites), the DNA repair protein Mre11, and the target of HU, ribonucleotide reductase (Rnr1). The genes encoding Rad proteins (*RAD7*, *RAD16*, and *RAD51*) all were upregulated in the *dnj4Δ* mutant but to a lesser extent than that in the WT (Fig. 4B). Overall, these data suggested that the mutant is unable to respond to DNA damage at the transcriptional level.

**FIG 5:**
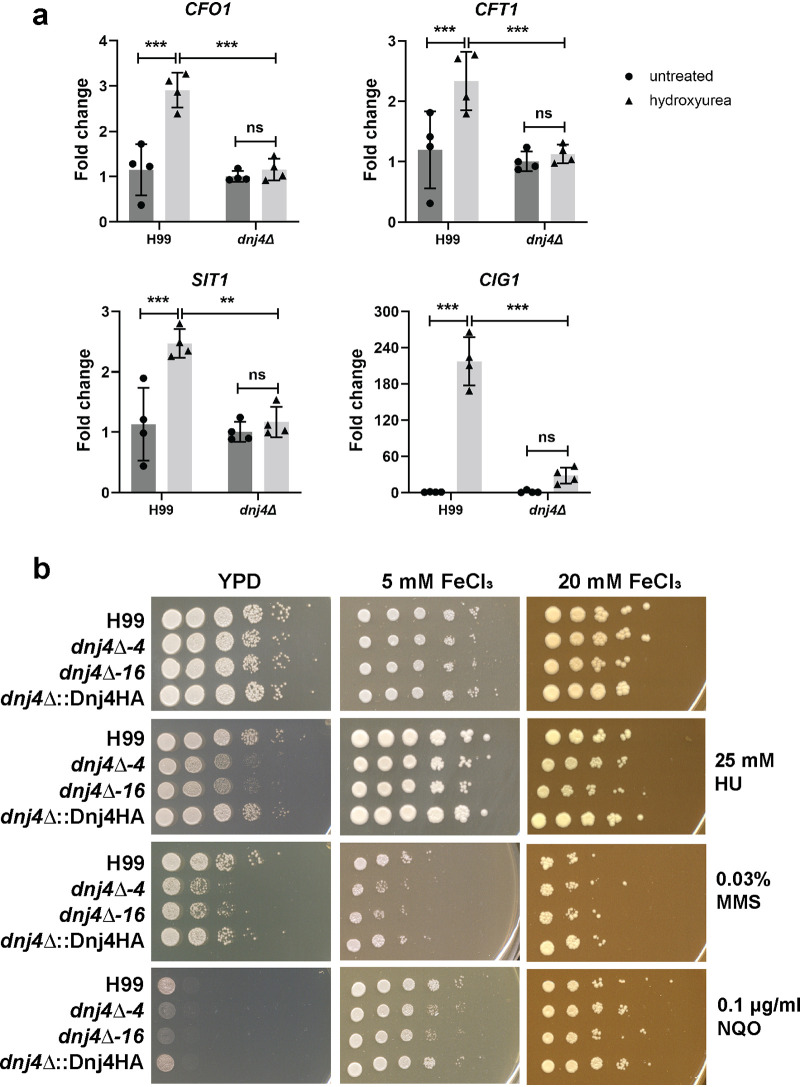
Dnj4 is required for iron homeostasis in response to hydroxyurea. (a) The changes in the transcript levels of iron acquisition genes (*CFO1*, *CFT1*, and *SIT1*) and the gene for the putative hemophore (*CIG1*) in response to HU were confirmed in the wild type (H99) and mutant (*dnj4Δ*) using RT-qPCR. Bars represent the means from four biological replicates, and error bars show the standard deviations. Statistical significance was determined using a two-way ANOVA and Tukey’s multiple comparisons (ns, not significant; **, *P* < 0.01; ***, *P* < 0.005). (b) Spot assays of serially diluted wild-type (H99), *dnj4Δ*, and complemented (*dnj4Δ*::Dnj4HA) strains are shown. The growth defect in the *dnj4Δ* mutants in the presence of HU was rescued by supplementation with excess iron at the indicated concentrations. Plates were incubated at 30°C for 2 to 5 days and scanned. Representative images of three independent spot assays are shown.

Interestingly, HU also induced the expression of iron uptake genes, including the putative hemophore Cig1 as the most upregulated gene upon HU treatment in the WT (Data Set S1). Similarly, siderophore transporters as well as the high-affinity iron uptake system (Cft1 and Cfo1) were significantly upregulated in the WT but not in the *dnj4Δ* mutant (Fig. 5a). Several enzymes that are required for DNA repair, including ribonucleotide reductase, require iron as an essential cofactor (30). Therefore, we hypothesized that an inability to obtain sufficient iron as cofactors contribute to the hypersensitivity of the *dnj4Δ* mutant to DNA damage. We examined the impact of iron on the susceptibility to DNA damage and found that excess iron restored WT-like growth in the *dnj4Δ* mutants in the presence of HU and NQO as well as partial restoration on MMS (Fig. 5b). This result is consistent with a role for Dnj4 in the regulation of iron uptake functions in response to DNA damage.

### Dnj4 is required for *in vitro* virulence factor production.

**FIG 6:**
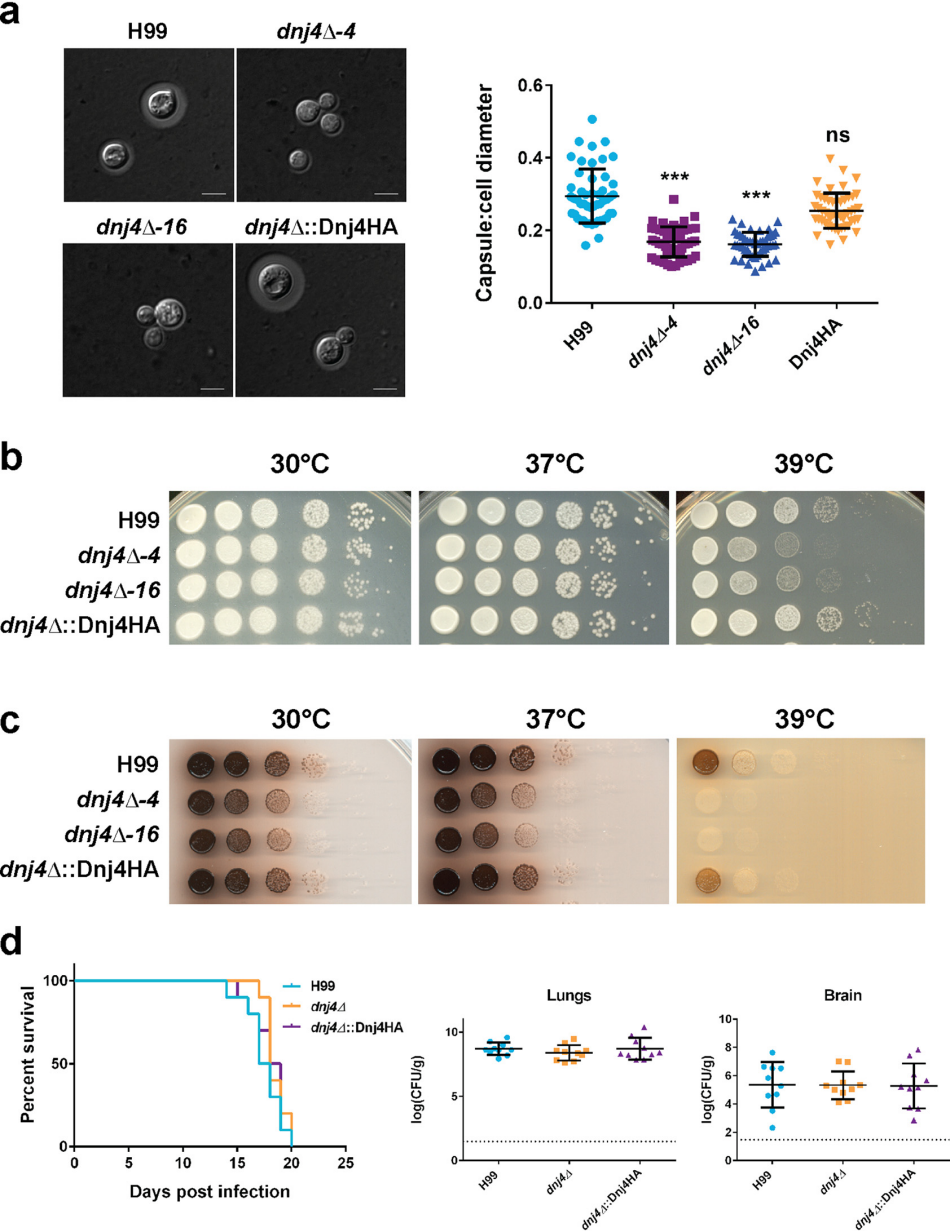
Dnj4 influences the elaboration of virulence factors *in vitro* but does not impact virulence *in vivo*. The elaboration of the three major virulence factors was examined in the wild type (H99), the *dnj4Δ* mutants (*dnj4Δ-4* and *dnj4Δ-16*), and the complement (*dnj4Δ*::Dnj4HA). (a) The capsule thickness and cell diameter were measured after 48 h of capsule induction for 50 cells in each strain. The quantification of the ratio of capsule thickness to cell diameter for each cell is shown as an individual point. The means and standard deviations are shown for each strain, and significance was determined using a one-way ANOVA with Tukey’s multiple comparisons (***, *P* < 0.005). (b and c) Spot assays of the indicated strains serially diluted and plated on YPD (b) and l-DOPA agar (c), with incubation at 30°C, 37°C, and 39°C as indicated. Spot assays shown are representative of three independent replicates. (d) Mice infected with the wild-type, mutant, and complemented strains all succumbed to infection between 14 and 21 days postinoculation. There was no significant difference in the survival of mice infected with different strains as determined with a log rank test. The numbers of CFU recovered from lung and brain tissue were quantified. Each dot represents the CFU recovered from one mouse, and the mean and standard deviation for each group is indicated. The dashed line indicates the limit of detection for the determination of CFU numbers in this experiment. There were no statistically significant differences between groups determined using Mann-Whitney U tests.

The regulation of iron uptake genes prompted us to evaluate elaboration of one of the major virulence factors in C. neoformans, the polysaccharide capsule, that is responsive to iron availability. When capsule production was induced in low-iron medium, the *dnj4Δ* mutants elaborated smaller capsules than the WT and complemented strains (Fig. 6a). The two other major virulence factors in C. neoformans, thermotolerance and melanin production, were also tested in the *dnj4Δ* mutants. The *dnj4Δ* mutants grew robustly at 37°C on YPD; however, their growth was slower than that of the WT and complemented strains at 39°C (Fig. 6b). A subtle decrease in melanin production was observed in the strains lacking *DNJ4* when grown on media supplemented with l-DOPA. No melanin production was observed in the deletion mutants at 39°C, although growth was severely decreased at this temperature (Fig. 6c). The elaboration of these three virulence factors *in vitro* was reduced in the *dnj4Δ* mutants.

### *DNJ4* is dispensable for virulence in a mouse model of cryptococcosis.

Since the *dnj4Δ* mutants had impaired elaboration of the three major virulence factors, we tested virulence in an intranasal murine model of cryptococcosis. Ten mice were inoculated with each strain and monitored for weight loss and disease symptoms. There were no differences in the survival of mice inoculated with the *dnj4Δ* mutant compared to the WT or complement (Fig. 6d). The mutant also proliferated and disseminated well in the mouse model, and fungal cells were recovered from the brain and lungs in numbers similar to those of the WT and complement strain (Fig. 6d). Fungal cells were also recovered from the blood of mice inoculated with each of the strains, but the mice infected with the *dnj4Δ* mutant had lower fungal burdens in the blood than mice inoculated with the WT or complemented strains (Fig. S5). Despite this difference, we concluded overall that Dnj4 does not play a major role in virulence, as there were no differences in fungal burden in other systemic organs (Fig. S5).

10.1128/mbio.03273-21.5 FIG S5 Dnj4 does not contribute to dissemination in a mouse model of cryptococcosis. Organs were recovered from mice infected with the wild-type (H99), *dnj4Δ* mutant, and complemented (*dnj4Δ*::Dnj4HA) strains and the CFUs recovered from blood (a), the spleen (b), the kidney (c), and the liver (d) were quantified. Each dot represents the CFUs recovered from one mouse and the mean and standard deviation for each group is indicated. The dashed line indicates the limit of detection for the determination of CFUs in this experiment. The statistical significance of differences between groups were assessed using Mann-Whitney U-tests (*, *P* < 0.05). Download FIG S5, SVG file, 0.2 MB. Copyright © 2021 Horianopoulos et al. 2021 Horianopoulos et al. https://creativecommons.org/licenses/by/4.0/ This content is distributed under the terms of the Creative Commons Attribution 4.0 International license.

### C. neoformans Dnj4 binds S. cerevisiae histones and interferes with histone chaperoning in S. cerevisiae.

**FIG 7:**
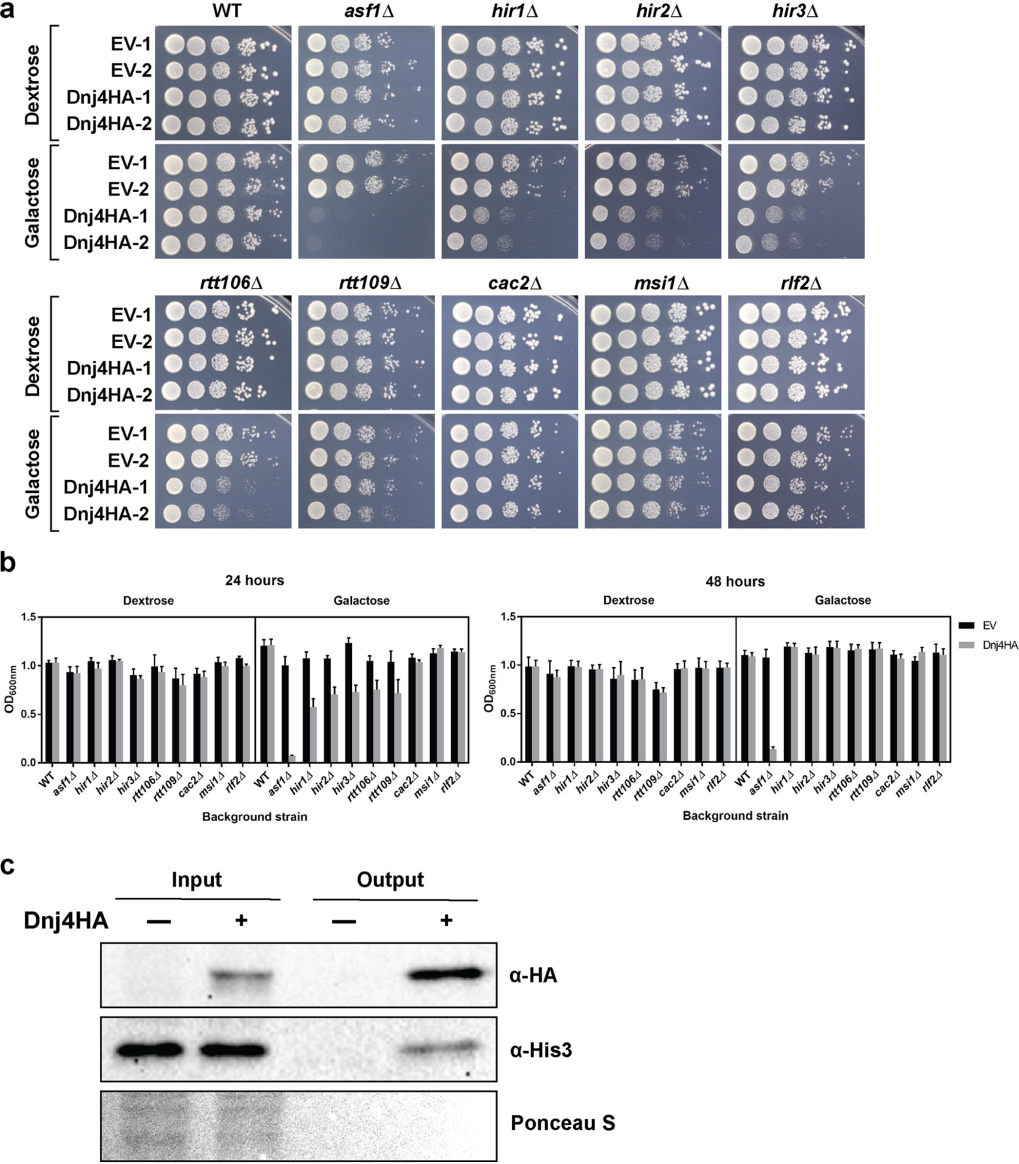
Dnj4 expression interferes with histone chaperoning in S. cerevisiae. S. cerevisiae WT and indicated mutant strains were transformed with a galactose-inducible promoter expressing Dnj4HA from C. neoformans or an empty vector (EV) control. Strains pregrown in YNB with raffinose were serially diluted and spotted on solid YNB (a) or inoculated in liquid YNB supplemented with either 2% dextrose or galactose (b) to control Dnj4HA expression and evaluate the impact of Dnj4HA on the growth of these strains. The bars in panel b represent the means from six replicates, and the error bars indicate the standard deviations. (c) The ability of C. neoformans Dnj4HA to bind S. cerevisiae histones was evaluated using coimmunoprecipitation and immunoblotting against the bait (Dnj4HA) and the prey (His3).

The human ortholog of Dnj4, DNAJC9, binds histones competitively with a central histone chaperone, Asf1, which is conserved from yeast to humans (22, 31). Furthermore, DNAJC9 allows histones to bypass Asf1-dependent pathways for interactions with DNA (22). To explore conservation of this interaction with Dnj4, we conditionally expressed CnDnj4HA in S. cerevisiae WT and histone chaperone mutants and characterized the resulting impact on growth. Strikingly, we found that expression of CnDnj4HA in an *asf1Δ* mutant caused a dramatic growth defect, whereas the growth of the WT was not impacted (Fig. 7a). Interestingly, mutants in the HIR complex (*hir1Δ*, *hir2Δ*, and *hir3Δ*) and an *rtt106Δ* mutant also had decreased growth upon expression of CnDnj4HA; however, this was not as dramatic as the defect in the *asf1Δ* mutant (Fig. 7a). Furthermore, in liquid SC medium lacking uracil (−Ura) supplemented with galactose to induce expression, the *asf1Δ* mutant expressing CnDnj4HA did not recover by 48 h of growth, further supporting the evidence that this mutation is synthetically lethal in the presence of CnDnj4HA (Fig. 7b). Because S. cerevisiae lacks an ortholog for Dnj4, we hypothesized that CnDnj4HA binds S. cerevisiae histones but that S. cerevisiae lacks the additional machinery to allow CnDnj4HA to facilitate Asf1-independent histone chaperoning. Therefore, in the background of mutants lacking key histone chaperones, CnDnj4HA would bind histones and disrupt their entry into other histone chaperoning pathways. In support of this idea, we verified that CnDnj4HA interacted with S. cerevisiae histone 3 using coimmunoprecipitation after 6 h of CnDnj4HA induction with 2% galactose (Fig. 7c). This result demonstrated that CnDnj4HA bound S. cerevisiae histones and that this interaction was likely interfering with the growth of mutants already impaired in histone chaperoning.

## DISCUSSION

The roles of J domain cochaperones in histone chaperoning were uncharacterized until recent proteomic approaches revealed that the human DNAJC9 protein integrates the heat shock response and histone chaperoning (22, 32). The ortholog of DNAJC9, which we designated Dnj4, in C. neoformans interacts with histones 3 and 4 as well as an Hsp70 (Hsp71-like protein, CNAG_01727). DNAJC9 is essential in most human cell lines (22); however, it is dispensable for routine growth in C. neoformans, allowing phenotypic characterization of a complete deletion mutant. The mutant lacking *DNJ4* had an impaired transcriptional response to DNA damage, hypersensitivity to DNA-damaging agents, and increased amounts of DNA damage. Together, these data suggest that the chromatin dynamics required to protect DNA and respond to damage are impaired in the absence of this histone chaperone, as has similarly been shown for the core histone chaperone Asf1 in S. cerevisiae (29, 33). Although Dnj4 is conserved between Homo sapiens and C. neoformans, there is no ortholog in S. cerevisiae. In this context, it was intriguing that heterologous expression of CnDnj4HA disrupted endogenous histone chaperoning pathways, causing toxicity and poor growth in mutants lacking the chaperone Asf1 and members of the HIR complex. Our demonstration that Dnj4HA bound S. cerevisiae histone 3 suggested sequestration of histones to prevent their entry into histone chaperoning pathways, particularly in the absence of Asf1, which is not only a core histone 3 and 4 chaperone (34) but also a competitive binder of DNAJC9 for these histones (22).

Because histones are basic proteins (35), they have a strong affinity for nonspecific interactions with DNA and must be chaperoned from synthesis to entry into the nucleus and throughout their dynamic interactions with DNA (34, 36, 37). The ability to properly chaperone histones is paramount to an organism’s ability to respond to changing environments and to preserve genomic integrity, which is crucial for opportunistic fungal pathogens. Recently, the HIR complex subunit Hir1 was shown to coordinate the transcriptional response to sensing nutritional changes, namely, protein as a major nitrogen source through a role in controlling chromatin accessibility in C. albicans (38). Similarly, Asf1 influences transcriptional responses to stimuli, including nutrient availability (39) and DNA damage (29) in S. cerevisiae. These studies, in combination with our findings that *DNJ4* was required for a robust transcriptional response to DNA damage, support an emerging picture in which histone chaperones play broad roles in facilitating transcriptional responses to changing environments in fungi. Since knockouts of the orthologs of Dnj4 in other species have few phenotypic differences from the respective type strains in the absence of genotoxic stress, they have been poorly characterized. However, the ortholog of Dnj4 in S. pombe shows sensitivity to hydroxyurea in a genome-wide screen of deletion mutants (40), and the ortholog in C. albicans was recently predicted to be a cell cycle regulator and was shown to be sensitive to hydroxyurea (41). Therefore, although the sensitivity to genotoxic stress has previously been established in these other species, this is the first description of a J domain protein acting as a histone chaperone in the kingdom Fungi. Since Dnj4 is not essential in C. neoformans, it presents an opportunity to further study mechanisms of genome stability involving J domain cochaperones. Finally, further characterization of the role of Dnj4 and its interplay with other histone chaperones or modifying enzymes may uncover novel mechanisms for the maintenance of genome stability, which can be used to potentiate the effects of drugs that target genomic integrity and chromatin dynamics in fungi.

## MATERIALS AND METHODS

### Strains and media.

Cryptococcus neoformans var. *grubii* (serotype A) strain H99 was used for all mutant construction and as the wild type (WT) in all experiments described in this study. All strains were routinely maintained on YPD medium (1% yeast extract, 2% peptone, 2% dextrose; BD Difco, Franklin Lakes, NJ). All engineered strains used in this study, including the deletion mutants and strains expressing C-terminal GFP and HA fusion proteins (see Table S2 in the supplemental material), were generated using biolistic transformation of linear constructs that were prepared using either three-step overlap PCR (42) or FastCloning into the genomic safe haven locus as previously described (43, 44). The primers used for generation of the transformed constructs are listed in Table S3. All chemicals were obtained from Sigma-Aldrich (St. Louis, MO) unless otherwise specified.

10.1128/mbio.03273-21.8 TABLE S2 Strains generated for the characterization of Dnj4. For each strain generated for this study, the resistance marker used and the background strain which was transformed to construct it are indicated. Download Table S2, PDF file, 0.02 MB. Copyright © 2021 Horianopoulos et al. 2021 Horianopoulos et al. https://creativecommons.org/licenses/by/4.0/ This content is distributed under the terms of the Creative Commons Attribution 4.0 International license.

10.1128/mbio.03273-21.9 TABLE S3 Primers and plasmids used for strain construction in the characterization of Dnj4. The primer names and sequences used to generate each construct are listed. The templates listed in the third columns provide information on whether this part of the construct was amplified from H99 gDNA or from one of the plasmids. In the final column, the primer pair for each primer is specified. Download Table S3, PDF file, 0.02 MB. Copyright © 2021 Horianopoulos et al. 2021 Horianopoulos et al. https://creativecommons.org/licenses/by/4.0/ This content is distributed under the terms of the Creative Commons Attribution 4.0 International license.

### Phylogenetic analysis.

The full-length amino acid sequence of Dnj4 (CNAG_03487) was used to identify the closest orthologs in Homo sapiens (DNAJC9) and Mus musculus (Dnajc9) using BLASTp (https://blast.ncbi.nlm.nih.gov/Blast.cgi?PAGE=Proteins), and fungal orthologs were identified using FungiDB (45). All amino acid sequences were retrieved from UniProt (https://www.uniprot.org/). The fungal orthologs included in this analysis were from Ustilago maydis (UMAG_04512), C. albicans (C4_06590W_A), Neurospora crassa (NCU17061), and Schizosaccharomyces pombe (SPAC1071.09C). Amino acid sequences for each protein were aligned in MEGA X (46) using the ClustalW algorithm. The Le Gascuel amino acid replacement matrix (47) was used to generate a maximum likelihood tree with 500 bootstraps to evaluate the robustness of the nodes of the resultant tree. The histone binding domain (HBD) of each of these proteins was also determined based on similarity to the HBD of DNAJC9 and aligned (22).

### Dnj4-GFP localization.

A strain expressing a Dnj4-GFP fusion protein was constructed in the background of the *dnj4Δ* mutant and expressed in the genomic safe haven locus under its native promoter (Table S2) (43). The strain expressing Dnj4-GFP was grown overnight in YNB plus 0.5% glucose with or without 25 mM hydroxyurea. Importantly, expression of this fusion protein from the genomic safe haven (43) in the background of the mutant restored wild-type growth in hydroxyurea. Untreated cells were also heat shocked at either 37°C or 42°C for 30 min. All cells were stained in phosphate-buffered saline (PBS) with 5 μg/ml DAPI for 15 min. Heat-shocked cells were maintained at the heat shock temperature during DAPI staining. Cells were washed once in PBS to remove extracellular dye and imaged using a Zeiss Plan-Apochromat 100×/1.46 oil lens on a Zeiss Axioplan 2 microscope. Images were obtained using an ORCA-Flash4.0 LT digital CMOS camera (Hamamatsu, Hamamatsu City, Japan). All fluorescent images were processed and nuclear fluorescence intensity was measured after background subtraction using Zen 3.0 software (Zeiss, Oberkochen, Germany).

### Growth and melanin assays.

Hypersensitivity to DNA-damaging agents was evaluated on YPD agar supplemented with 25 mM hydroxyurea (HU), 0.03% 4-nitroquinoline 1-oxide, or 0.1 μg/ml methyl methanesulfonate. To assess the rescue of sensitivity to DNA damage with iron overload, YPD agar was further supplemented with 5 mM or 20 mM FeCl_3_. For each condition tested and YPD agar controls, WT, *dnj4Δ* mutants, and *dnj4Δ*::Dnj4HA complemented cells were 10-fold serially diluted and spotted onto solid media starting at 10^5^ cells per 5-μl spot. The same procedure was used to test melanin formation on chemically defined media containing 0.1% l-asparagine, 0.1% dextrose, 3 mg/ml KH_2_PO_4_, 0.25 mg/ml MgSO_4_·7H_2_O, 1 μg/ml thiamine, 5 ng/ml biotin, and 0.2 mg/ml l-3,4-dihydroxyphenylalanine (l-DOPA). Plates were incubated at 30°C, 37°C, or 39°C for 2 to 5 days and scanned to evaluate differences in growth between strains.

### Protein extraction.

Overnight YPD cultures of WT and *dnj4Δ*::Dnj4HA strains were diluted 1:10 in fresh YPD in a final volume of 50 ml and grown for 6 h at 30°C with shaking to obtain log-phase cells. Protein extraction was performed as previously described (48, 49). Briefly, cells were flash frozen in liquid nitrogen and pulverized using a precooled mortar and pestle. Pulverized cells were collected and resuspended in lysis buffer containing 50 mM Tris-HCl, pH 7.5, 5 mM EDTA, 100 mM NaCl, 1% Triton X-100, and 1× EDTA-free protease inhibitor cocktail (Roche, Basel, Switzerland). The broken cells in lysis buffer were vortexed and sonicated in a Bioruptor Pico (Diagenode, Sparta, NJ) water bath sonicator at 4°C for five 30-s cycles with 1 min between cycles to solubilize proteins. Cell debris was removed by centrifugation at 13,500 rpm for 15 min, and the protein concentration in lysates was determined using the Pierce bicinchoninic acid protein assay kit (Thermo Fisher, Waltham, MA) by following the manufacturer’s instructions.

### Coimmunoprecipitation and mass spectrometry.

The proteins interacting with a Dnj4-HA fusion protein were identified as previously described (49). Briefly, 1.5 mg of protein lysate was added to 25 μl of Pierce anti-HA magnetic bead slurry (Thermo Fisher) and rotated for 2 h at 4°C. Magnetic beads were washed three times in Tris-buffered saline (TBS)  plus 0.05% Tween 20, eluted in 100 μl 50 mM NaOH, and neutralized using 50 μl 1 M Tris, pH 8.5. Eluted proteins were further concentrated by chloroform-methanol precipitation (50). Precipitated proteins were digested using RapiGest (Waters, Milford, MA) by following the manufacturer’s in-solution digest protocol and acidified using trifluoroacetic acid. Stop-and-go extraction (STAGE) tipping was performed as previously described for acidic solutions (51, 52). The resulting peptides were resuspended in sample buffer containing 2% acetonitrile and 0.1% formic acid.

Reverse-phase liquid chromatography (LC) was performed on 2 μl of each sample for peptide separation using an RSLCnano Ultimate 3000 system (Thermo Fisher Scientific). Peptides were loaded on an Acclaim PepMap 100 precolumn (100 μm by 2 cm, C_18_, 5 μm, 100 Å; Thermo Fisher Scientific) with 0.07% trifluoroacetic acid at a flow rate of 20 μl/min for 3 min. Analytical separation of peptides was performed on an Acclaim PepMap RSLC column (75 μm by 50 cm, C_18_, 2 μm, 100 Å; Thermo Fisher Scientific) at a flow rate of 300 nL/min. The solvent composition was gradually changed over 94 min from 96% solvent A (0.1% formic acid) and 4% solvent B (80% acetonitrile, 0.1% formic acid) to 10% solvent B within 2 min, to 30% solvent B within the next 58 min, to 45% solvent B within the following 22 min, and to 90% solvent B within the last 12 min. All solvents and acids were Optima grade for liquid chromatography mass spectrometry (LC-MS; Thermo Fisher Scientific). Eluting peptides were on-line ionized by nano-electrospray ionization (nESI) using the Nanospray Flex Ion source (Thermo Scientific) at 1.5 kV (liquid junction) and transferred into a Q Exactive HF mass spectrometer (Thermo Fisher Scientific). Full scans in a mass range of 300 to 1,650 *m/z* were recorded at a resolution of 30,000 followed by data-dependent top 10 HCD fragmentation at a resolution of 15,000 (dynamic exclusion enabled). The LC-MS method programming and data acquisition were performed with the XCalibur 4.0 software (Thermo Fisher Scientific). The resultant RAW files were deposited in the PRIDE (53) partner repository with the data set identifier PXD028087.

MaxQuant 1.6.0.16 (54, 55) was used for protein identification and label-free quantification by searching MS/MS2 data against the C. neoformans var. *grubii* H99 protein database from UniProt (UP000010091; downloaded 19 October 2018). Protein identification was conducted in MaxQuant using a false discovery rate (FDR) of 0.01, and quantification was conducted for proteins with a minimum of two peptides. The default settings of MaxQuant were used with the addition of label-free quantification selected in group-specific parameters. The results of the MaxQuant analysis were further processed and statistically analyzed using Perseus 1.6.0.7 (56). Statistical significance of the enriched proteins in the strain expressing the fusion protein was evaluated using a one-sided *t* test with a false discovery rate of 0.05 in Perseus.

### Immunoblotting.

The eluates from coimmunoprecipitation using Dnj4HA as bait and 15 μg of protein from whole-cell lysate were subjected to electrophoresis in each well of a 15% SDS-PAGE experiment with subsequent transfer of proteins onto a polyvinylidene difluoride membrane (GE Healthcare, Boston, MA) using wet transfer at 70 V for 3 h. Membranes were blocked in TBST with 5% skim milk and incubated with 1:10,000 monoclonal mouse anti-HA (cat no. 26183; Thermo Fisher) or 1:5,000 rabbit anti-acetyl histone 3 (cat no. 06-942) as primary antibodies followed by 1:5,000 goat anti-mouse horseradish peroxidase (HRP) (cat no. 170-6516; Bio-Rad, Hercules, CA) or 1:5,000 goat anti-rabbit HRP (cat no. 170-6515; Bio-Rad). All immunoblots were visualized using chemiluminescence (GE Healthcare).

### RNA extraction.

Overnight YPD cultures of WT and *dnj4Δ* mutant strains were diluted 1:10 with fresh YPD in a final volume of 15 ml and grown to log phase for 3 h at 30°C with shaking. After 3 h, cells were collected and resuspended in 15 ml of either fresh YPD as a control or YPD supplemented with 25 mM HU and incubated for an additional hour with or without treatment. Cells were harvested, frozen in liquid nitrogen, and stored at −80°C. RNA was extracted using a Qiagen RNeasy kit (Qiagen, Hilden, Germany) by following the manufacturer’s instructions for mechanical disruption of yeast with bead beating. Contaminating DNA was removed using the Turbo DNase kit (Ambion, Austin, TX) by following the manufacturer’s instructions.

### RNA sequencing.

Samples containing 5 μg of DNase-treated RNA extracted from three biological replicates of HU-treated and control WT and *dnj4Δ* mutant were submitted to Genewiz (South Plainfield, NJ) for RNA sequencing. Briefly, the mRNA was enriched through poly(A) selection, cDNA was synthesized, and adapters were ligated to the ends of cDNA fragment and sequenced using Illumina HiSeq 2 × 150-bp sequencing. Raw reads were trimmed for adapter content and aligned to the C. neoformans var. *grubii* transcriptome (CNA3; GCA_000149245.3) using STAR (57). Each sample mapped >90% uniquely aligned reads, and transcript-specific hit counts were determined with geneCounts. Significantly differentially expressed genes were determined in R using DESeq2 using a *P* value cutoff of 0.05 and a minimum of a 2-fold change (58).

To identify significantly overrepresented functional groups within the up- and downregulated genes, H99 gene identifiers (IDs) were first converted to gene IDs from the genome of C. neoformans strain JEC21 using FungiDB for compatibility to KEGG database annotations. Gene set enrichment analysis (GSEA) was then used to identify enriched pathways based on a ranked gene list generated from DESeq2 output and pathway information from the KEGG database (59). GSEA was carried out at 10,000 permutations and included gene sets between 10 and 400. An enrichment map was then used to visualize the results at a Benjamini Hochberg FDR value of 0.25 and *P* value cutoff of 0.05 for all comparisons (60).

### RT-qPCR.

cDNA was synthesized using the high-capacity cDNA reverse transcription kit (Applied Biosystems, Foster City, CA) using oligo(dT) primer. cDNA was used as the template for quantitative reverse transcription-PCR (RT-qPCR) using Green-2-Go qPCR Mastermix (Bio Basic Amherst, NY) with low ROX and the primers listed in Table S4. Thermocycling and signal detection were performed using a 7500 Fast real-time PCR system (Applied Biosystems). The relative expression of each target gene was quantified using the 2^−ΔΔCT^ method (61) and normalized to both *ACT1* and *GAPDH.* Statistical significance of the differences in expression between treatments and strains was determined using a two-way analysis of variance (ANOVA) with Tukey’s correction for multiple tests in GraphPad 7 (GraphPad Software, San Diego, CA).

10.1128/mbio.03273-21.10 TABLE S4 Primer sequences for RT-qPCR confirmation of the RNA-Seq data. Download Table S4, PDF file, 0.02 MB. Copyright © 2021 Horianopoulos et al. 2021 Horianopoulos et al. https://creativecommons.org/licenses/by/4.0/ This content is distributed under the terms of the Creative Commons Attribution 4.0 International license.

### Comet assay.

Spheroplasts were generated as previously described, with minor modifications (62). Briefly, overnight cultures grown in YPD were diluted in 15 ml fresh YNB plus 1 M NaCl to an optical density at 600 nm (OD_600_) of 0.1 and grown in a 30°C shaking incubator until they reached an OD_600_ of 0.4 (approximately 8 h). Cells were collected, washed three times with 0.5 M NaCl–50 mM EDTA, pH 8, resuspended in 5 ml 5% β-mercaptoethanol, and incubated at 37°C for shaking at 70 rpm for 1 h. Cells were pelleted and washed three times in spheroplasting buffer (1 M sorbitol, 10 mM EDTA, 100 mM sodium citrate, pH 5.5) and resuspended in 2 ml spheroplasting buffer with 20 mg of lysing enzymes from *Trichoderma harzianum* and incubated at 37°C with shaking at 70 rpm overnight.

Spheroplasts were mixed with four volumes of 1% low-melting-point agarose, pipetted onto glass slides precoated with 1% agarose, and solidified for 15 min on ice. Glass slides were immersed in lysis buffer (1% sarcosyl, 0.5 M EDTA, 10 mM Tris-HCl, pH 10) and incubated at 4°C overnight. Slides were washed three times in electrophoresis buffer (30 mM NaOH, 2 mM EDTA) and subjected to electrophoresis at 0.6 V/cm for 25 min as previously described (25). Slides were rinsed in water, stained in 2.5 μg/ml propidium iodide for 20 min, and rinsed once more before visualization at 40× with a Zeiss Axioplan 2 microscope. The percentage of tail DNA was calculated using CometScore 2.0.0.38, and statistical significance was determined in GraphPad 7 (GraphPad) using a one-way ANOVA with Tukey’s multiple comparisons.

### Evaluation of capsule production.

Capsule production was induced in low iron capsule inducing media (CIM) as previously described (63). Cells were grown overnight in YPD and washed in sterile low-iron water, and CIM was inoculated with 10^6^ cells/ml. Cells were grown for 48 h at 30°C under inducing conditions, and India ink was used as a negative stain to visualize the capsule. The cell diameter and capsule thickness were measured for 50 cells from each strain using ImageJ (64). The ratio of capsule thickness to cell diameter was calculated, and the statistical significance of the differences observed between strains was evaluated using a one-way ANOVA with Tukey’s multiple comparisons in GraphPad 7 (GraphPad).

### Virulence assay.

The WT, *dnj4Δ* mutants, and *dnj4Δ*::Dnj4HA complemented strains were grown overnight in YPD at 30°C. Cells were washed three times in sterile PBS (Gibco, Waltham, MA) and resuspended at 4 × 10^6^ cells/ml in PBS. These cell suspensions were used as the inocula for *in vivo* assessment of virulence. Ten female BALB/c mice (4 to 6 weeks old; Charles River Laboratories, ON, Canada) were inoculated with each strain by intranasal instillation with 50 μl of cell suspension (2 × 10^5^ cells per mouse). The infected mice were monitored and weighed daily postinoculation to assess the occurrence of disease symptoms. Mice displaying significant weight loss and signs of morbidity were euthanized by CO_2_ anoxia. Cardiac blood was retrieved and organs were excised, weighed, and homogenized in two volumes of PBS using a MixerMill MM400 (Retsch, Haan, Germany). Tenfold serial dilutions of blood and homogenized tissue were plated on YPD agar plates supplemented with 50 μg/ml chloramphenicol and incubated at 30°C for 2 days, and CFU numbers were determined. Statistical significance in the survival assay was determined using a log rank test and the statistical significance between strains in fungal burdens was determined using Mann-Whitney U tests in GraphPad 7 (GraphPad). All experiments with mice were conducted in accordance with the guidelines of the Canadian Council on Animal Care and approved by the University of British Columbia’s Committee on Animal Care (protocol A17-0117).

### Heterologous expression of Dnj4HA in Saccharomyces cerevisiae.

RNA was extracted from the Dnj4HA-expressing complement and used to produce cDNA with the high-capacity cDNA reverse transcription kit (Applied Biosystems) using oligo(dT) primer. Dnj4HA was amplified from the cDNA to remove introns and cloned into the pAG416GAL-ccdB plasmid (65) using FastCloning (44) and the primers listed in Table S3. This plasmid as well as the empty vector control was transformed into S. cerevisiae *asf1Δ*, *cac2Δ*, *hir1Δ*, *hir2Δ*, *hir3Δ*, *msi1Δ*, *rlf2Δ*, *rtt106Δ*, and *rtt109Δ* deletion mutants and the background WT strain BY4741 obtained from the Kan deletion library (66) using the lithium acetate transformation as previously described (67). The impact of Dnj4HA on the growth of these mutants was tested by overnight growth in YNB with amino acids lacking uracil (YNB −Ura) with 2% raffinose, dilution to an OD_600_ of 0.1, serial dilution, and plating on solid YNB −Ura with either 2% galactose to induce expression or 2% dextrose to repress expression or, alternatively, inoculation at an OD_600_ of 0.03 into liquid YNB with the same carbon sources.

The ability of Dnj4HA to bind S. cerevisiae histones was tested by inducing Dnj4HA expression for 6 h in YNB −Ura plus 2% galactose and extracting total protein using bead beating for 2 cycles of 1 min with 1 min on ice between cycles in lysis buffer (50 mM Tris-HCl, pH 7.5, 5 mM EDTA, 100 mM NaCl, 1% Triton X-100, and 1× EDTA-free protease inhibitor cocktail [Roche]). A volume of 500 μg of protein lysate was added to 20 μl of Pierce anti-HA magnetic bead slurry (Thermo Fisher) and rotated for 2 h at 4°C. Magnetic beads were washed three times in TBS plus 0.05% Tween 20, eluted in 80 μl 50 mM NaOH, and neutralized using 40 μl 1 M Tris, pH 8.5. The eluted proteins and total lysate controls were analyzed by immunoblotting.

### Data availability.

The RAW files generated from the affinity purification-mass spectrometry experiments have been deposited in the PRIDE partner repository with the data set identifier PXD028087.

The RNA-Seq data have been deposited to Gene Expression Omnibus with the identifier GSE182879.
