# Supplementary material for: A J Domain Protein Functions as a Histone Chaperone to Maintain Genome Integrity and the Response to DNA Damage in a Human Fungal Pathogen
Source: mBio. 2021 Dec 21;12(6):e03273-21. doi: 10.1128/mbio.03273-21 (PMC8689522; doi:10.1128/mbio.03273-21)
Supplement: TABLE S1 [file mbio.03273-21-st001.pdf]

**Table S1. Proteins detected through AP-MS.**

| Sig | p-value | Δlog2(LFQ intensity) | Peptides | Protein IDs | Gene name |
|---|---|---|---|---|---|
| + | 5.19E-06 | 11.21124 | 30 | J9VQF6 | CNAG_03487 |
| + | 0.006791 | 3.645885 | 5 | J9VSI7;J9VMZ0 | CNAG_04828; CNAG_06745 |
| + | 0.005758 | 3.184739 | 12 | J9VUB1 | CNAG_01648 |
| | 0.170033 | 1.761897 | 13 | J9VGW3 | CNAG_03053 |
| | 0.055281 | 1.716628 | 7 | J9W2U5 | CNAG_06222 |
| | 0.02249 | 1.687939 | 16 | J9W2T4 | CNAG_06208 |
| | 0.070845 | 1.625262 | 7 | T2BN17;J9VQF1 | CNAG_03482 |
| | 0.016342 | 1.559745 | 9 | T2BPD2;J9VH85 | CNAG_04028 |
| | 0.07124 | 1.484854 | 6 | O94746 | FRR1 |
| | 0.204175 | 1.438976 | 15 | J9VM00 | CNAG_02332 |
| | 0.214502 | 1.430317 | 17 | J9VIA4 | CNAG_07323 |
| | 0.099972 | 1.41882 | 18 | J9VSX3 | CNAG_04676 |
| + | 0.009254 | 1.403735 | 20 | J9VUR9 | CNAG_04441 |
| + | 0.00971 | 1.276826 | 5 | J9VID8 | CNAG_02754 |
| | 0.057977 | 1.244466 | 6 | J9VWJ8 | CNAG_01586 |
| + | 0.007101 | 1.213212 | 5 | J9VGY9 | CNAG_03015 |
| | 0.129249 | 1.186124 | 10 | J9VFG4 | CNAG_00703 |
| | 0.043871 | 1.170925 | 8 | J9VGP4 | CNAG_03817 |
| | 0.052742 | 1.115067 | 9 | J9VNN9 | CNAG_05762 |
| | 0.039776 | 1.10874 | 12 | J9VI77 | CNAG_03602 |
| | 0.11229 | 1.084763 | 10 | J9VYN9 | CNAG_01564 |
| + | 0.000994 | 1.078419 | 4 | J9VUF9 | CNAG_01896 |
| + | 0.000616 | 1.040138 | 12 | J9VSC8 | CNAG_04448 |
| | 0.010166 | 1.023802 | 23 | J9VSJ2 | CNAG_02234 |
| | 0.075634 | 1.008042 | 13 | J9VZ02 | CNAG_06447 |
| | 0.13384 | 0.96412 | 9 | J9W3D8 | CNAG_06468 |
| + | 0.003544 | 0.960417 | 15 | J9VXL7 | CNAG_03198 |
| | 0.105557 | 0.959571 | 7 | J9VI18 | CNAG_06770 |

| | | | | | |
|---|---|---|---|---|---|
| | 0.027421 | 0.95159 | 6 | J9VG91 | CNAG_00232 |
| | 0.023913 | 0.932739 | 16 | J9W2G5 | CNAG_06095 |
| | 0.05221 | 0.929726 | 8 | J9VVT7 | CNAG_06535 |
| | 0.12795 | 0.914527 | 12 | J9VPF5 | CNAG_05199 |
| | 0.117124 | 0.888323 | 12 | J9VR33 | CNAG_03510 |
| | 0.010578 | 0.887249 | 20 | J9VXE8 | CNAG_01884 |
| | 0.057264 | 0.875807 | 8 | J9VK44 | CNAG_02880 |
| + | 0.005458 | 0.869083 | 25 | J9VZ70 | CNAG_01727 |
| | 0.205617 | 0.865039 | 8 | J9VZ91 | CNAG_01752 |
| | 0.149323 | 0.857751 | 15 | J9VJ21 | CNAG_03891 |
| | 0.026284 | 0.847984 | 12 | J9VFV3 | CNAG_03747 |
| | 0.095051 | 0.838823 | 5 | J9VPE0 | CNAG_03127 |
| | 0.245524 | 0.835236 | 21 | J9VXY1;T2BN03 | CNAG_03345 |
| | 0.080973 | 0.819573 | 12 | J9VY95 | CNAG_04601 |
| | 0.067577 | 0.807992 | 11 | J9VI11;J9VLX7 | CNAG_04099;<br>CNAG_07561 |
| | 0.035896 | 0.786723 | 12 | J9VF13 | CNAG_00771 |
| | 0.185324 | 0.784758 | 12 | J9VY31 | CNAG_04694 |
| | 0.061381 | 0.778378 | 4 | J9VL49 | CNAG_01181 |
| | 0.048384 | 0.76556 | 5 | J9VM77 | CNAG_00666 |
| | 0.161746 | 0.747513 | 4 | J9VQA5 | CNAG_03438 |
| | 0.270378 | 0.735867 | 9 | J9VV17 | CNAG_07637 |
| | 0.011853 | 0.731933 | 24 | J9VX05 | CNAG_05555 |
| | 0.314683 | 0.731675 | 14 | J9VXX2 | CNAG_03335 |
| | 0.118064 | 0.719212 | 9 | J9VW13 | CNAG_04609 |
| | 0.01466 | 0.712123 | 16 | J9VFY8 | CNAG_03780 |
| | 0.13445 | 0.709407 | 15 | J9VPP7 | CNAG_05918 |
| | 0.013293 | 0.681393 | 6 | J9VVM5 | CNAG_06273 |
| | 0.082007 | 0.678389 | 8 | J9VV89 | CNAG_01984 |
| + | 0.002086 | 0.67776 | 19 | J9VTC1 | CNAG_04726 |
| | 0.027252 | 0.676297 | 20 | J9W0I6 | CNAG_06231 |
| | 0.247786 | 0.667671 | 7 | J9VJJ1 | CNAG_00655 |

| | | | | |
|---|---|---|---|---|
| 0.0544 | 0.649733 | 16 | J9VPA5 | CNAG_04021 |
| 0.257224 | 0.643569 | 3 | J9VI08 | CNAG_06779 |
| 0.121581 | 0.641579 | 8 | J9VRV1 | CNAG_02485 |
| 0.08408 | 0.630775 | 23 | J9VP88 | CNAG_05750 |
| 0.047391 | 0.62037 | 17 | J9VHE4 | CNAG_00672 |
| 0.216769 | 0.605762 | 4 | J9VMQ3 | CNAG_00819 |
| 0.100006 | 0.603642 | 12 | J9VV35 | CNAG_06113 |
| 0.298438 | 0.592832 | 4 | J9VRV4 | CNAG_04488 |
| 0.078826 | 0.59055 | 16 | J9VM66 | CNAG_00656 |
| 0.102551 | 0.583615 | 7 | J9VHC1 | CNAG_04068 |
| 0.04806 | 0.566583 | 6 | J9VJC5 | CNAG_04011 |
| 0.055409 | 0.564863 | 17 | J9VD88 | CNAG_00116 |
| 0.358584 | 0.538815 | 27 | J9VWY4 | CNAG_01682 |
| 0.220579 | 0.530319 | 4 | J9VQC2 | CNAG_01323 |
| 0.159282 | 0.524274 | 6 | J9VF52 | CNAG_00821 |
| 0.162141 | 0.523745 | 7 | J9VTA9 | CNAG_01486 |
| 0.335573 | 0.515884 | 14 | J9VSX5 | CNAG_02100 |
| 0.207559 | 0.515799 | 12 | J9VUD6 | CNAG_05904 |
| 0.117259 | 0.507196 | 18 | T2BNZ0;J9VL75 | CNAG_01152 |
| 0.207508 | 0.495678 | 9 | J9VR87 | CNAG_04969 |
| 0.188395 | 0.489453 | 11 | J9VQI0 | CNAG_03283 |
| 0.124906 | 0.487586 | 8 | J9VHL5 | CNAG_02811 |
| 0.168839 | 0.485163 | 4 | J9VQ73 | CNAG_02838 |
| 0.052991 | 0.481211 | 40 | J9VEL7 | CNAG_00334 |
| 0.225865 | 0.479369 | 15 | J9VQI1 | CNAG_02144 |
| 0.183348 | 0.476416 | 7 | J9VUU8 | CNAG_02359 |
| 0.057155 | 0.474703 | 13 | J9VQB5 | CNAG_02209 |
| 0.163865 | 0.46405 | 17 | J9VJ71 | CNAG_05232 |
| 0.093583 | 0.457177 | 12 | J9VR32 | CNAG_06919 |
| 0.211179 | 0.443084 | 8 | J9VQ03 | NOG2 |
| 0.073987 | 0.442049 | 4 | J9W045 | CNAG_04840 |

| | | | | |
|---|---|---|---|---|
| 0.263145 | 0.441008 | 14 | J9VIB2 | CNAG_03641 |
| 0.102813 | 0.437215 | 19 | J9VP67 | CNAG_05725 |
| 0.363648 | 0.435662 | 7 | J9VGF1 | CNAG_03722 |
| 0.071703 | 0.427607 | 13 | J9VK13 | CNAG_00788 |
| 0.224637 | 0.426995 | 21 | J9VPU9 | CNAG_02418 |
| 0.272668 | 0.423717 | 6 | J9VT09 | TIF6 |
| 0.250542 | 0.419518 | 12 | J9W146 | CNAG_06472 |
| 0.304598 | 0.40639 | 5 | J9VMB8 | CNAG_00705 |
| 0.332602 | 0.403764 | 15 | J9VU24 | CNAG_01568 |
| 0.073425 | 0.401307 | 11 | J9VTE3 | CNAG_01170 |
| 0.128066 | 0.392965 | 14 | J9VLR6 | CNAG_02437 |
| 0.102776 | 0.37061 | 16 | J9VP81 | CNAG_05976 |
| 0.327971 | 0.361752 | 12 | J9VUH9 | CNAG_01679 |
| 0.133386 | 0.3616 | 31 | J9VF99 | CNAG_00640 |
| 0.246577 | 0.354199 | 13 | J9VV50 | CNAG_07839 |
| 0.316941 | 0.352383 | 10 | J9VKH7 | CNAG_00062 |
| 0.18775 | 0.34621 | 17 | J9VSC4 | CNAG_02330 |
| 0.236079 | 0.341773 | 8 | J9VTA4 | CNAG_01480 |
| 0.323393 | 0.340783 | 11 | J9VXP0 | CNAG_05600 |
| 0.175971 | 0.339411 | 10 | J9VN98 | CNAG_03675 |
| 0.295801 | 0.337119 | 22 | J9VMN2 | CNAG_02331 |
| 0.219836 | 0.326951 | 7 | J9VNW6 | CNAG_06633 |
| 0.013352 | 0.325425 | 11 | J9VU89 | CNAG_01628 |
| 0.136567 | 0.324863 | 10 | J9VN14 | CNAG_02720 |
| 0.091575 | 0.322049 | 4 | J9W225 | CNAG_01976 |
| 0.277714 | 0.317369 | 21 | J9VKV8 | CNAG_03739 |
| 0.242485 | 0.316062 | 11 | J9VM09 | CNAG_02545 |
| 0.333635 | 0.315643 | 16 | J9VW40 | CNAG_06605 |
| 0.206507 | 0.306024 | 4 | J9VU28 | CNAG_05800 |
| 0.294316 | 0.297562 | 8 | J9VFJ7 | NOP7 |
| 0.057745 | 0.29469 | 15 | J9VKK2 | CNAG_01153 |

| | | | | |
|---|---|---|---|---|
| 0.374109 | 0.288954 | 12 | J9VMM1 | CNAG_00779 |
| 0.127968 | 0.280938 | 6 | J9VVA3 | CNAG_03303 |
| 0.041838 | 0.276526 | 14 | J9VDR3 | CNAG_00034 |
| 0.124011 | 0.259057 | 14 | J9VLJ8 | CNAG_03944 |
| 0.385306 | 0.258945 | 21 | J9VMP7 | CNAG_00809 |
| 0.415297 | 0.255477 | 7 | J9VLV3 | CNAG_05131 |
| 0.394155 | 0.25466 | 7 | J9VSB6 | CNAG_02340 |
| 0.319856 | 0.249435 | 15 | J9VKA5 | CNAG_06971 |
| 0.416317 | 0.24943 | 15 | J9VJ06 | CNAG_00447 |
| 0.340568 | 0.244918 | 11 | J9VT96 | CNAG_01224 |
| 0.268328 | 0.243305 | 30 | J9W0B1 | CNAG_04762 |
| 0.296576 | 0.242959 | 7 | J9VU67 | CNAG_01780 |
| 0.19337 | 0.239982 | 11 | J9VKN9 | CNAG_01332 |
| 0.22523 | 0.23199 | 9 | J9VHK8 | CNAG_00741 |
| 0.274267 | 0.217419 | 29 | J9VLP5 | RPS1 |
| 0.295352 | 0.212087 | 11 | J9VK98 | CNAG_06811 |
| 0.407452 | 0.211974 | 3 | J9VW88 | CNAG_06274 |
| 0.382559 | 0.21187 | 5 | J9VPZ5 | CNAG_02917 |
| 0.161493 | 0.202354 | 9 | J9VRH1;J9VRZ1 | CNAG_04523; CNAG_06699 |
| 0.34712 | 0.195929 | 13 | J9VFW4 | CNAG_00104 |
| 0.2793 | 0.191785 | 13 | J9VKK7 | CNAG_01148 |
| 0.376708 | 0.145754 | 9 | J9VJ67 | CNAG_00513 |
| 0.345823 | 0.137866 | 26 | J9VMD3 | CNAG_02928 |
| 0.440054 | 0.128824 | 13 | J9VQY3 | CNAG_01136 |
| 0.383145 | 0.124744 | 60 | J9VQZ9 | CNAG_01117 |
| 0.193334 | 0.121035 | 11 | J9VZS2 | CNAG_01990 |
| 0.428886 | 0.118269 | 15 | J9W0Q4 | CNAG_04580 |
| 0.330329 | 0.117644 | 12 | J9VME1 | CNAG_00730 |
| 0.413728 | 0.114658 | 15 | Q6SSJ3 | ILV2 |
| 0.37582 | 0.10258 | 15 | J9VSL1 | CNAG_04799 |
| 0.457792 | 0.096921 | 12 | J9VUC9 | CNAG_01664 |

| | | | | |
|---|---|---|---|---|
| 0.420703 | 0.074982 | 6 | J9VK51 | CNAG_01300 |
| 0.445722 | 0.074802 | 18 | J9VXH5 | CNAG_04883 |
| 0.466368 | 0.060356 | 12 | J9VSI8 | CNAG_02239 |
| 0.435722 | 0.050437 | 5 | J9VW77 | CNAG_06502 |
| 0.465742 | 0.044727 | 16 | J9VER4 | CNAG_00410 |
| 0.466215 | 0.044125 | 4 | J9VSM6 | CNAG_01413 |
| 0.475718 | 0.033535 | 13 | J9VSC0 | CNAG_02335 |
| 0.476942 | 0.027402 | 19 | J9VFX9;T2BQ66 | CNAG_03771 |
| 0.466219 | 0.027163 | 7 | J9VZA0 | CNAG_01761 |
| 0.471182 | 0.024227 | 22 | J9VY34;J9VV25 | CNAG_06101; CNAG_06102 |
| 0.484185 | 0.023465 | 21 | J9VN41 | CNAG_03606 |
| 0.483904 | 0.021673 | 9 | J9VHX2 | CNAG_02664 |
| 0.480677 | 0.02044 | 13 | J9VTK1 | CNAG_01577 |
| 0.491696 | 0.008144 | 75 | J9VF80 | CNAG_07373 |
| 0.493546 | 0.00738 | 7 | J9VXK4 | CNAG_05556 |
| 0.504844 | -0.0046 | 11 | J9W0E5 | CNAG_06182 |
| 0.503687 | -0.0063 | 19 | J9VVH4 | CNAG_02099 |
| 0.533959 | -0.01623 | 15 | J9VQ82 | CNAG_02257 |
| 0.522415 | -0.01865 | 9 | J9VYL9 | CNAG_01544 |
| 0.639712 | -0.02514 | 5 | J9VUI3 | CNAG_04340 |
| 0.564127 | -0.03807 | 48 | J9VGR3 | CNAG_04082 |
| 0.524784 | -0.03845 | 9 | J9VLH1 | CNAG_05031 |
| 0.605874 | -0.03879 | 9 | T2BP43;J9VIP1 | CNAG_00305 |
| 0.518461 | -0.04417 | 12 | J9VR81 | CNAG_01018 |
| 0.521357 | -0.05965 | 18 | J9VTL0 | CNAG_01091 |
| 0.550128 | -0.06302 | 6 | J9VW63 | CNAG_06630 |
| 0.535862 | -0.06883 | 2 | J9VUN6 | CNAG_01751 |
| 0.66084 | -0.07468 | 39 | J9VWK6 | CNAG_06400 |
| 0.580534 | -0.07646 | 8 | T2BMM3;T2BMP7;J9VQE9 | CNAG_02174 |
| 0.52323 | -0.08203 | 5 | J9VNL9 | CNAG_06563 |
| 0.603821 | -0.08702 | 17 | J9VMF7 | CNAG_07400 |

| | | | | |
|---|---|---|---|---|
| 0.543589 | -0.08758 | 12 | J9VEY5 | CNAG_00512 |
| 0.540672 | -0.10565 | 9 | J9VXL5 | CNAG_01961 |
| 0.6663 | -0.1212 | 9 | J9VNN1 | CNAG_01120 |
| 0.600283 | -0.12623 | 10 | J9VVZ9 | CNAG_03577 |
| 0.546233 | -0.1298 | 11 | J9VTC9 | CNAG_04715 |
| 0.62945 | -0.1307 | 25 | Q8TG24 | MET3 |
| 0.637145 | -0.13126 | 3 | J9VK09 | CNAG_02916 |
| 0.608594 | -0.13175 | 11 | J9VLB4 | CNAG_04976 |
| 0.5893 | -0.16542 | 21 | J9VP17 | CNAG_05907 |
| 0.807711 | -0.17801 | 10 | J9VY17 | CNAG_04709 |
| 0.575049 | -0.19265 | 12 | J9VVV4 | CNAG_04687 |
| 0.927041 | -0.2052 | 17 | J9VZI7 | CNAG_04445 |
| 0.716897 | -0.22946 | 6 | J9VVL6 | CNAG_03435 |
| 0.919968 | -0.23306 | 26 | J9VW24 | CNAG_06443 |
| 0.642017 | -0.23928 | 14 | J9VDY5 | CNAG_00111 |
| 0.553561 | -0.24392 | 15 | J9W469 | CNAG_05602 |
| 0.704436 | -0.24519 | 14 | J9VVK6 | CNAG_04800 |
| 0.793719 | -0.25031 | 24 | J9VGW8 | CNAG_07363 |
| 0.784053 | -0.25678 | 30 | J9W2J0 | CNAG_06125 |
| 0.78468 | -0.26238 | 10 | J9VXK6 | CNAG_01951 |
| 0.696284 | -0.26421 | 16 | J9VMD6 | CNAG_07382 |
| 0.744939 | -0.26604 | 8 | J9VQ41 | CNAG_01414 |
| 0.874635 | -0.27137 | 18 | J9VIJ6 | CNAG_00256 |
| 0.661579 | -0.27403 | 9 | T2BNJ3;J9VX99 | CNAG_01820 |
| 0.928228 | -0.2782 | 16 | J9VN00 | CNAG_02736 |
| 0.800389 | -0.28739 | 13 | J9W1J2 | CNAG_01733 |
| 0.675017 | -0.29308 | 5 | J9VXQ3 | CNAG_03249 |
| 0.938786 | -0.29504 | 7 | J9VT08 | CNAG_03315 |
| 0.743222 | -0.29666 | 7 | J9VUE0 | CNAG_05909 |
| 0.693502 | -0.31363 | 3 | J9VMC4 | CNAG_02938 |
| 0.657201 | -0.31865 | 24 | J9VJF3 | CNAG_07810 |

| | | | | |
|---|---|---|---|---|
| 0.977331 | -0.32002 | 17 | J9VYP1 | RPS0 |
| 0.880347 | -0.32136 | 9 | J9VYC1 | CNAG_03507 |
| 0.856257 | -0.32761 | 16 | J9W358 | CNAG_06377 |
| 0.889059 | -0.34254 | 16 | J9VXW3 | CNAG_07851 |
| 0.767374 | -0.35596 | 10 | J9VVQ2 | CNAG_06301 |
| 0.733446 | -0.36523 | 14 | J9VXI8 | CNAG_03168 |
| 0.759659 | -0.37536 | 7 | T2BN71;J9VMC0 | CNAG_02943 |
| 0.821365 | -0.37999 | 30 | J9W3X8 | CNAG_05465 |
| 0.840756 | -0.38015 | 14 | J9VPD8 | CNAG_05179 |
| 0.758885 | -0.38856 | 26 | J9VWX1 | CNAG_05900 |
| 0.764948 | -0.38902 | 9 | J9VKM9 | CNAG_00108 |
| 0.835066 | -0.39929 | 11 | J9VZA8 | CNAG_05365 |
| 0.749596 | -0.41211 | 12 | J9VUD2 | CNAG_02502 |
| 0.854453 | -0.41685 | 35 | J9VVA4 | CNAG_06150 |
| 0.983614 | -0.42743 | 10 | J9VHG6 | CNAG_00697 |
| 0.948561 | -0.43684 | 17 | J9W1R2 | CNAG_01840 |
| 0.922071 | -0.43791 | 14 | J9VI76 | CNAG_02815 |
| 0.851431 | -0.44809 | 14 | J9VGV0 | CNAG_03072 |
| 0.887934 | -0.4561 | 30 | J9VNZ3 | CNAG_05070 |
| 0.986138 | -0.46875 | 13 | J9VMJ3 | CNAG_02377 |
| 0.627203 | -0.46933 | 24 | J9VMY2 | TIF32 |
| 0.990558 | -0.47567 | 9 | J9VEX3 | CNAG_00747 |
| 0.728222 | -0.47694 | 6 | J9VZT6 | CNAG_05525 |
| 0.864523 | -0.48145 | 18 | J9VTV3 | CNAG_00992 |
| 0.792361 | -0.49799 | 9 | J9VXV6 | CNAG_06012 |
| 0.945318 | -0.50698 | 22 | J9VFR5 | CNAG_00058 |
| 0.870295 | -0.51927 | 15 | J9VEG9 | TIF34 |
| 0.935583 | -0.52068 | 11 | J9W0N0 | CNAG_04605 |
| 0.730521 | -0.52739 | 12 | J9VGS7 | CNAG_00418 |
| 0.987591 | -0.52744 | 18 | P48465 | CNAG_00483 |
| 0.933499 | -0.52981 | 15 | J9VVP1 | CNAG_03459 |

| | | | | |
|---|---|---|---|---|
| 0.645317 | -0.53931 | 6 | J9VPF2 | CNAG_05814 |
| 0.991207 | -0.54144 | 15 | J9VP27 | CNAG_03920 |
| 0.982675 | -0.54151 | 15 | J9VL11 | CNAG_03787 |
| 0.931256 | -0.56878 | 10 | J9VGH0 | CNAG_07346 |
| 0.736821 | -0.57544 | 14 | J9VPI4 | CNAG_07676 |
| 0.896303 | -0.58916 | 47 | J9VQK7 | CNAG_07746 |
| 0.829933 | -0.60368 | 7 | J9VN60 | TIF35 |
| 0.881617 | -0.60925 | 14 | J9VMS4 | CNAG_02814 |
| 0.964705 | -0.61451 | 14 | J9VKA9 | CNAG_07004 |
| 0.858691 | -0.6392 | 15 | J9VZ71 | CNAG_04304 |
| 0.965444 | -0.65227 | 14 | J9VKD0 | CNAG_01435 |
| 0.946424 | -0.65748 | 12 | J9VS17 | CNAG_05235 |
| 0.958657 | -0.67296 | 6 | J9VZD9 | CNAG_01818 |
| 0.931126 | -0.67418 | 12 | J9VH03 | CNAG_03000 |
| 0.914012 | -0.67608 | 11 | J9VZD5 | CNAG_01813 |
| 0.889421 | -0.67682 | 18 | J9VXN5 | CNAG_03225 |
| 0.98991 | -0.68027 | 4 | J9VLE6;J9VXH6 | CNAG_00370; CNAG_01920 |
| 0.918258 | -0.69723 | 14 | J9VM05 | CNAG_02326 |
| 0.882566 | -0.70322 | 71 | J9VXF1 | CNAG_01890 |
| 0.914352 | -0.71011 | 13 | J9VT12 | CNAG_03320 |
| 0.974625 | -0.72797 | 6 | Q85SZ4 | COII |
| 0.983684 | -0.72979 | 10 | J9W2N9 | CNAG_06153 |
| 0.876791 | -0.73835 | 39 | J9W0K1 | CNAG_04640 |
| 0.805707 | -0.7452 | 17 | J9VL88 | CNAG_07347 |
| 0.928029 | -0.78035 | 20 | J9VD92 | CNAG_00121 |
| 0.94082 | -0.78148 | 24 | J9VV75 | CNAG_03263 |
| 0.816058 | -0.80776 | 13 | J9VRE8 | CNAG_00935 |
| 0.966724 | -0.80891 | 5 | J9W241 | CNAG_01991 |
| 0.981641 | -0.82178 | 16 | J9VX38 | CNAG_01744 |
| 0.999845 | -0.82553 | 7 | J9VLD5 | CNAG_01083 |
| 0.940727 | -0.82969 | 13 | J9W0X6 | CNAG_06381 |

| | | | | |
|---|---|---|---|---|
| 0.891138 | -0.83411 | 15 | J9VW35 | CNAG_06600 |
| 0.99449 | -0.83577 | 13 | J9VRA9 | CNAG_04990 |
| 0.754657 | -0.90471 | 14 | J9VU59 | CNAG_04189 |
| 0.948649 | -0.91145 | 17 | J9VXU0 | CNAG_03299 |
| 0.998244 | -0.91498 | 10 | J9VU38 | CNAG_01745 |
| 0.986668 | -0.93201 | 29 | J9VHP1 | CNAG_02974 |
| 0.991196 | -0.93417 | 11 | J9VTW1 | CNAG_01492 |
| 0.83024 | -0.95108 | 26 | J9VI50 | CNAG_00147 |
| 0.903249 | -0.95214 | 39 | J9VJJ6;T2BNJ0 | CNAG_06840 |
| 0.800952 | -0.95402 | 14 | J9VMC5 | NIP1 |
| 0.923715 | -0.97146 | 22 | J9VLI9 | CNAG_00417 |
| 0.952463 | -1.01829 | 13 | J9VM27 | CNAG_00622 |
| 0.915793 | -1.0416 | 14 | J9VMZ2 | CNAG_02748 |
| 0.97791 | -1.05325 | 18 | J9VN22 | CNAG_02710 |
| 0.909608 | -1.13769 | 40 | J9VVE7 | CNAG_02129 |
| 0.962039 | -1.15732 | 8 | J9VL79 | CNAG_03853 |
| 0.990061 | -1.16036 | 17 | J9W2Q3 | CNAG_06168 |
| 0.841987 | -1.17192 | 12 | J9VRJ5 | CNAG_00891 |
| 0.990127 | -1.17426 | 4 | J9VGJ7 | CNAG_00316 |
| 0.993058 | -1.21589 | 16 | J9VH45 | CNAG_00565 |
| 0.982339 | -1.23568 | 16 | J9VFK3 | CNAG_03629 |
| 0.995247 | -1.30352 | 14 | J9VNN2 | CNAG_04948 |
| 0.885567 | -1.32113 | 27 | J9VVR3 | CNAG_06123 |
| 0.930314 | -1.43734 | 13 | J9VHS2 | CNAG_00785 |
| 0.937818 | -1.45229 | 32 | J9VL23 | CNAG_07445 |
| 0.9195 | -1.46792 | 42 | J9VRN8;T2BP70 | CNAG_05753 |
| 0.825829 | -1.48313 | 35 | J9VVY8 | CNAG_06585 |
| 0.977134 | -1.55844 | 30 | J9VR74 | CNAG_03554 |
| 0.994282 | -1.74059 | 24 | J9VU15 | CNAG_01558 |
| 0.958955 | -1.82457 | 63 | J9VTZ1 | CNAG_05759 |
| 0.986622 | -1.86017 | 11 | J9VS53 | CNAG_04362 |

| | | | | |
|---|---|---|---|---|
| 0.926568 | -2.05636 | 15 | J9VNQ3 | CNAG_03824 |
| 0.959973 | -2.2436 | 11 | J9VME3 | CNAG_02918 |
| 0.999593 | -2.33031 | 12 | J9VKH0 | CNAG_00057 |
| 0.969897 | -2.47201 | 25 | J9VQN8 | CNAG_03358 |
| 0.97958 | -2.88252 | 27 | J9VNJ0 | CNAG_01164 |
| 0.978621 | -3.23567 | 31 | Q059G6 | TPS2 |
| 0.950763 | -3.80292 | 26 | J9VW78 | CNAG_01464 |

The Δlog2(LFQ intensity) is the average change in Label Free Quantification (LFQ) intensity from 3 replicates between the pull down with the Dnj4-HA tagged strain and the WT control. The p-value is derived from a one-sided t-test (FDR = 0.05) performed in Perseus and the significance based on this test is indicated in the first column.
