# Supplementary material for: A J Domain Protein Functions as a Histone Chaperone to Maintain Genome Integrity and the Response to DNA Damage in a Human Fungal Pathogen
Source: mBio. 2021 Dec 21;12(6):e03273-21. doi: 10.1128/mbio.03273-21 (PMC8689522; doi:10.1128/mbio.03273-21)
Supplement: TABLE S2 [file mbio.03273-21-st002.pdf]

Table S2 Strains generated for the characterization of Dnj4.

Strain	Genotype	Background
*dnj4*Δ-4	*dnj4::NEO*	H99
*dnj4*Δ-16	*dnj4::NEO*	H99
*Dnj4*Δ::Dnj4HA	*Dnj4HA::NAT*	*dnj4*Δ-16
Dnj4-GFP	*dnj4::NEO*<br>*Dnj4GFP::HYG*	*dnj4*Δ-16

For each strain generated for this study, the resistance marker used and the background strain which was transformed to construct it are indicated.
