# Supplementary material for: A J Domain Protein Functions as a Histone Chaperone to Maintain Genome Integrity and the Response to DNA Damage in a Human Fungal Pathogen
Source: mBio. 2021 Dec 21;12(6):e03273-21. doi: 10.1128/mbio.03273-21 (PMC8689522; doi:10.1128/mbio.03273-21)
Supplement: TABLE S3 [file mbio.03273-21-st003.pdf]

Table S3 Primers and plasmids used for strain construction in the characterization of Dnj4.

Primer name	Sequence 5'-3'	Plasmids and templates	Primer pair
Dnj4-1	GGAAAGAGGAGCCCTCATAGC	H99	Dnj4-2
Dnj4-2	AGCTCACATCCTCGCAGCACTAACACCAGCTGCAGACG	H99	Dnj4-1
Dnj4-3	CGTCTGCAGCTGGTGTTAGTGCTGCGAGGATGTGAGCT	pJAF1	Dnj4-4
Dnj4-4	TGGGGCCTTGGTACTGTCTCGAAGAGATGTAGAAACTA	pJAF1	Dnj4-3
Dnj4-5	TAGTTTCTACATCTCTTCGAGACAGTACCAAGGCCCCA	H99	Dnj4-6
Dnj4-6	GCCATCACAGATCAGGTGAG	H99	Dnj4-5
Dnj4HA-1	GGAAAGAGGAGCCCTCATAGC	H99	Dnj4HA-2
Dnj4HA-2	TCAGGGACATCGTAAGGGTAGGCCTTGGACTTTTTTGATTTCTTTG	H99	Dnj4HA-1
Dnj4HA-3	ATCAAAAAAGTCCAAGGCCTACCCTTACGATGTCCCTGATTACG	Mrj1HA	Dnj4HA-4
Dnj4HA-4	CTAGGCCTTGGACTTTTTTGAGATGTAGAAACTAGCTTCCTGG	Mrj1HA	Dnj4HA-3
Dnj4HA-5	GCTAGTTTCTACATCTCAAAAAAGTCCAAGGCCTAGATATTTG	H99	Dnj4HA-6
Dnj4HA-6	GCCATCACAGATCAGGTGAG	H99	Dnj4HA-5
Dnj4-GFPiF	GAATTGGGTACCGGGGAAAGAGGAGCCCTCATAGC	H99	Dnj4-GFPiR
Dnj4-GFPiR	TCGCCCTTGCTCACGGCCTTGGACTTTTTTGATTTCTTTG	H99	Dnj4-GFPiF
Dnj4-GFPvF	GTGAGCAAGGGCGAGGAGCT	pHD58	Dnj4-GFPvR
Dnj4-GFPvR	CCGGTACCCAATTCGCCCTATAG	pHD58	Dnj4-GFPvF
pRS416VF	GGATCCACTAGTTCTAGAATCC	pRS416	pRS416VR
pRS416VR	CGATACCGTCGACCTCGAG	pRS416	pRS416VF
Dnj4HAIF	TAGAACTAGTGGATCCATGGACGACGCCGATCCTATC	Dnj4HA cDNA	Dnj4HAIR
Dnj4HAIR	GAGGTCGACGGTATCGTCAAGCGTAATCAGGGACATCG	Dnj4HA cDNA	Dnj4HAIF

The primer names and sequences used to generate each construct are listed. The templates listed in the third columns provide information on whether this part of the construct was amplified from H99 gDNA or from one of the plasmids. In the final column, the primer pair for each primer is specified.
