# Supplementary material for: A J Domain Protein Functions as a Histone Chaperone to Maintain Genome Integrity and the Response to DNA Damage in a Human Fungal Pathogen
Source: mBio. 2021 Dec 21;12(6):e03273-21. doi: 10.1128/mbio.03273-21 (PMC8689522; doi:10.1128/mbio.03273-21)
Supplement: TABLE S4 [file mbio.03273-21-st004.pdf]

Table S4 Primer sequences for RT-qPCR confirmation of the RNA-Seq data.

Primer name	Sequence 5’-3’
ACT1qF	CACCATTGGTAACGAGCGATTC
ACT1qR	TGGTAGTACCACCAGACATGAC
GAPDHqF	GCCGTAGGCAAGGTCATTC
GAPDHqR	CCTTCAACTCAGGGCTCTC
POL4qF	ACTGTGGACGAGAAGTTGTGG
POL4qR	CCATCGGTGTCATCCCTAGTG
RFA1qF	CGCACAAGTAAAGGACGAGC
RFA1qR	TCACACCACCAACCACTACC
RNR1qF	CAGCCCAATGAAGCAAGTGAC
RNR1qR	CTACATTGCAACGCCGCTTC
RAD7qF	ATGCCTGCGTAACCTCACAG
RAD7qR	TCGCTAAGCTCATGACCCTTC
RAD16qF	GGCTATGGACCGTATTCACCG
RAD16qR	CCCAGTGCAGAATCCGAATC
RAD51qF	TACATCGACACGGAAGGCAC
RAD51qR	TCATGGCACTCGCTTGTACC
REV1qF	AGGTGTTGAGATGGATGAGGG
REV1qR	TCACCTCATCCCTCATTTGCC
MRE11qF	ACGAGGAAGAGGAAGAGGAGG
MRE11qR	GACTTAGCTGGCGTTCTTGC
CFO1qF	GGACCTTGGCCGCTCAA
CFO1qR	CAAGCGCGCCAATCG
CFT1qF	GGATATAAATCCGCCGCTCTT
CFT1qR	TTCCTTGGCCCTCTTCTCTTC
SIT1qF	GCCGCCATTTGGACCAA
SIT1qR	GCACGGAGGAGGTCGTTGTA
CIG1qF	CATCTGGTTCTAAGCTCTCTGC
CIG1qR	GAAGATACAGACTCGTGGTCG
